# Synthesis, characterization, crystal structure and Hirshfeld surface analysis of a hexa­hydro­quinoline derivative: *tert*-butyl 4-([1,1′-biphen­yl]-4-yl)-2,6,6-trimethyl-5-oxo-1,4,5,6,7,8-hexa­hydro­quinoline-3-carboxyl­ate

**DOI:** 10.1107/S2056989022007022

**Published:** 2022-07-14

**Authors:** Sema Öztürk Yıldırım, Mehmet Akkurt, Gökalp Çetin, Rahime Şimşek, Ray J. Butcher, Ajaya Bhattarai

**Affiliations:** a Department of Physics, Faculty of Science, Eskisehir Technical University, Yunus Emre Campus 26470 Eskisehir, Turkey; b Department of Physics, Faculty of Science, Erciyes University, 38039 Kayseri, Turkey; c Department of Pharmaceutical Chemistry, Faculty of Pharmacy, Erzincan Binali Yıldırım University, 24100 Erzincan, Turkey; d Department of Pharmaceutical Chemistry, Faculty of Pharmacy, Hacettepe University, 06100 Sıhhiye-Ankara, Turkey; e Department of Chemistry, Howard University, Washington DC 20059, USA; f Department of Chemistry, M.M.A.M.C (Tribhuvan University), Biratnagar, Nepal; Vienna University of Technology, Austria

**Keywords:** crystal structure, 1,4-di­hydro­pyridine ring, cyclo­hexene ring, quinoline ring system, van der Waals inter­actions, Hirshfeld surface analysis, disorder

## Abstract

The title compound, C_29_H_33_NO_3_, crystallizes with three mol­ecules (*A*, *B* and *C*) in the asymmetric unit. They differ in the twist of the phenyl and benzene rings of the 1,1′-biphenyl ring with respect to the plane of the 1,4-di­hydro­pyridine ring. In all three mol­ecules, the 1,4-di­hydro­pyridine ring adopts a distorted boat conformation. The cyclo­hexene ring has an envelope conformation in mol­ecules *A* and *B*, while it exhibits a distorted half-chair conformation for both the major and minor components in the disordered mol­ecule *C*. In the crystal, mol­ecules are linked by C—H⋯O and N—H⋯O hydrogen bonds, forming layers parallel to (100) defining *R* ^1^_4_(6) and *C*(7) graph-set motifs. Additional C—H⋯π inter­actions consolidate the layered structure. Between the layers, van der Waals inter­actions stabilize the packing, as revealed by Hirshfeld surface analysis. The greatest contributions to the crystal packing are from H⋯H (69.6% in *A*, 69.9% in *B*, 70.1% in *C*), C⋯H/H⋯C (20.3% in *A*, 20.6% in *B*, 20.3% in *C*) and O⋯H/H⋯O (8.6% in *A*, 8.6% in *B*, 8.4% in *C*) inter­actions.

## Abstract 2

C—H⋯O and N—H⋯O hydrogen bonds connect mol­ecules in the crystal, generating layers parallel to the (100) plane with 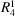 (6) and *C*(7) graph-set motifs. C—H⋯π inter­actions help to reinforce this layered mol­ecular structure.

## 1. Chemical context

Chronic diseases are among the most common causes of death in the world, accompanied by difficulties and costs in treatment and health care. Therefore, preventing or treating chronic diseases is of paramount importance (Raghupathi & Raghupathi, 2018 ▸). Recent advances have shown that many diseases such as cancer, atherosclerosis or neurodegenerative diseases are triggered by inflammation (Furman *et al.*, 2019 ▸). Based on these findings, regulating inflammatory mediators and pathways has been suggested as a treatment strategy (Kany *et al.*, 2019 ▸).

Inflammatory stimuli that cause chronic inflammation initiate the production of inflammatory mediators such as inter­leukin-1β (IL-1β), inter­leukin-6 (IL-6) and tumor necrosis factor-α (TNF-α) as a result of the activation of signaling pathways. Receptors activated by inflammatory mediators induce chronic inflammation by various signaling pathways (nuclear factor κ-B (NF-KB), Janus kinase (JAK), signal transducer and activator of transcription (STAT). Inhibiting these pathways may be a promising approach for the treatment of chronic diseases associated with inflammation (Chen *et al.*, 2018 ▸).

Nifedipine, the first drug with a 1,4-di­hydro­pyridine (1,4-DHP) ring, was introduced as a therapeutic agent as a result of intensive studies. The success of nifedipine as an anti­hypertensive drug has led to further studies and the discovery of other 1,4-DHP derivatives (De Luca *et al.*, 2019 ▸). Numerous compounds were obtained through modifications with respect to the 1,4-DHP ring. These studies also uncovered the idea of obtaining hexa­hydro­quinoline derivatives by condensation of the 1,4-DHP scaffold with the cyclo­hexane ring system (Bladen *et al.*, 2014 ▸). In recent years, it has been found that 1,4-DHP and quinoline analogs have the potential to inhibit inflammation mediators and pathways, along with various other pharmacological activities (Costa *et al.*, 2010 ▸; Längle *et al.*, 2015 ▸; Kim *et al.*, 2018 ▸; Çetin *et al.*, 2022 ▸). 
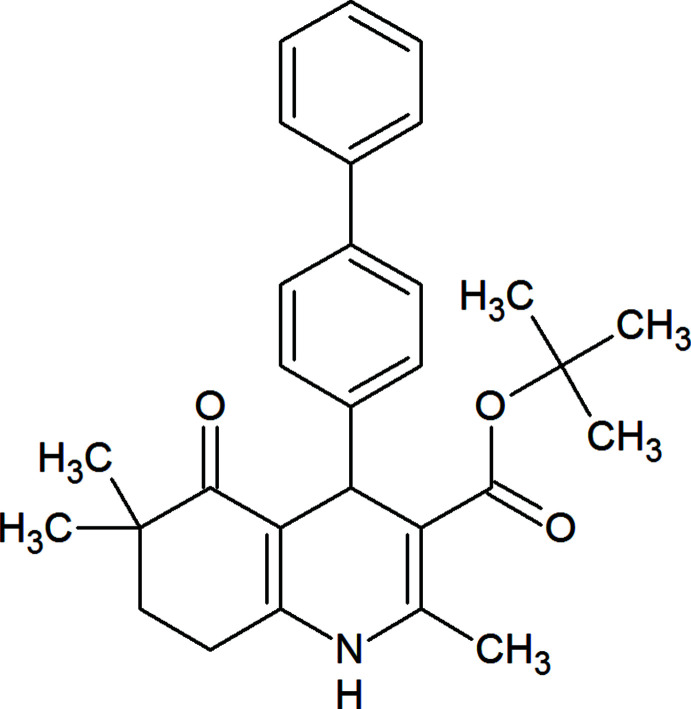


In the current study, *tert*-butyl 4-[(1,1′-biphen­yl)-4-yl]-2,6,6-trimethyl-5-oxo-1,4,5,6,7,8-hexa­hydro­quinoline-3-carboxyl­ate was obtained by condensation of the 1,4-DHP ring with a substituted cyclo­hexane ring using a modified Hantzsch method. The mol­ecular structure of the compound was confirmed by spectroscopic methods such as IR, ^1^H NMR, ^13^C NMR, and its composition by elemental analysis. In addition, single-crystal X-ray analysis was performed to elucidate the crystal structure of the compound. Independent of the current study, biological activity studies of the title compound are ongoing.

## 2. Structural commentary

**Figure 1:**
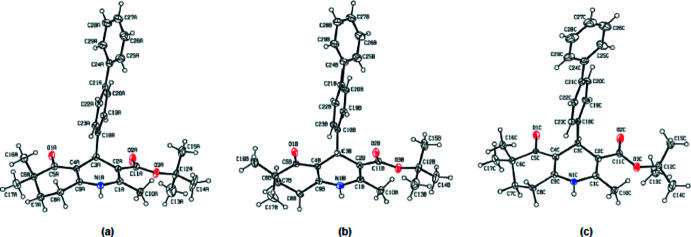
Views from the same direction of the three mol­ecules in the asymmetric unit of the title compound, with displacement ellipsoids for the non-hydrogen atoms drawn at the 30% probability level. (*a*) Mol­ecule *A*, (*b*) mol­ecule *B*, and (*c*) mol­ecule *C* (only the major component of the disorder is shown).

**Figure 2:**
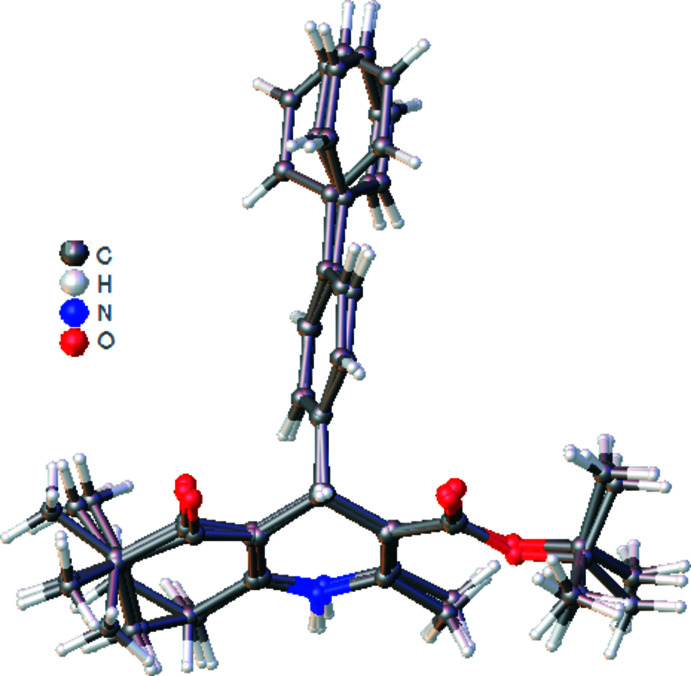
Overlay image of the three independent mol­ecules of the title compound. While the terminal phenyl rings of mol­ecules *A* and *B* coincide well, that of mol­ecule *C* is not in the same plane with them, and is approximately normal to them. Only the major component of the disorder in mol­ecule *C* is shown.

The asymmetric unit of the title compound (Fig. 1 ▸) contains three independent mol­ecules (denoted with suffixes *A*, *B* and *C*). They mainly differ in the twist of the phenyl (C24–C29) and benzene (C18–C23) rings of the 1,1′-biphenyl ring with respect to the plane of the 1,4-di­hydro­pyridine ring (N1/C1–C4/C9). The corresponding dihedral angles amount to 89.26 (16) and 75.83 (19)° in mol­ecule *A*, 88.34 (17) and 71.7 (2)° in mol­ecule *B*, and 89.38 (17) and 83.6 (3)° in mol­ecule *C*. The phenyl and benzene rings of the 1,1′-biphenyl ring make dihedral angles of 39.05 (19)° in *A*, 46.9 (2)° in *B*, and 33.5 (2)° in *C*. Fig. 2 ▸ shows an overlay plot of mol­ecules *A*, *B* and *C*, with an r.m.s. deviation of 0.725 Å. Except for the atoms of the minor part of the disordered mol­ecule *C* and the phenyl ring of the biphenyl group, the other atoms of mol­ecule *C* and all atoms of mol­ecules *A* and *B* are quite compatible and coincide with each other.

In all three mol­ecules, the 1,4-di­hydro­pyridine ring adopts a distorted boat conformation with puckering parameters (Cremer & Pople, 1975 ▸) *Q*_T_ = 0.269 (4) Å, θ = 104.5 (9)° and φ = 357.4 (9)° in *A*, *Q*_T_ = 0.257 (4) Å, θ = 73.1 (9)° and φ = 176.0 (9)° in *B*, and *Q*_T_ = 0.303 (4) Å, θ = 106.9 (8)° and φ = 356.2 (8)° in *C*. The cyclo­hexene ring (C4–C9) has an envelope conformation in mol­ecules *A* and *B* [the puckering parameters are *Q*_T_ = 0.430 (4) Å, θ = 49.3 (5)° and φ = 182.3 (7)° in *A*, and *Q*_T_ = 0.439 (4) Å, θ = 58.8 (5)° and φ = 179.9 (6)° in *B*], while the major and minor components of the disordered cyclo­hexene rings in *C* exhibit a distorted half-chair conformation, with puckering parameters of *Q*_T_ = 0.451 (9) Å, θ = 44.7 (12)° and φ = 161 (2)° for the major component, and of *Q*_T_ = 0.44 (2) Å, θ = 50 (3)° and φ = 206 (5)° for the minor component.

Bond lengths and angles in the three mol­ecules of the title compound are comparable with those of closely related structures detailed in section 5 (*Database survey*).

## 3. Supra­molecular features

**Figure 3:**
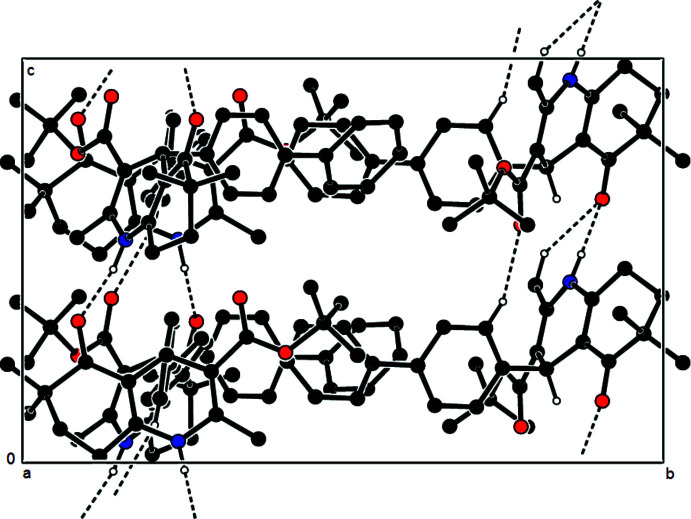
A view of the inter­molecular N—H⋯O and C—H⋯O inter­actions in the crystal structure of the title compound projected along [100]. Only the major component of the disordered mol­ecule *C* is shown.

**Figure 4:**
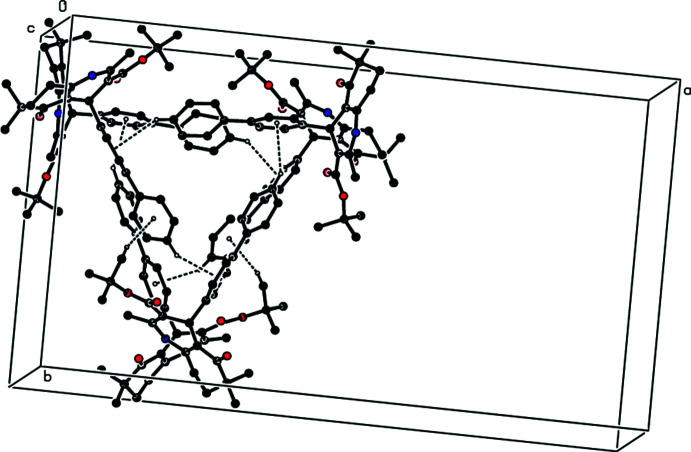
A general view of a part of the mol­ecular packing formed by C—H⋯π inter­actions in the crystal structure of the title compound. Only the major component of the disordered mol­ecule *C* is shown.

In the crystal, mol­ecules are linked by C—H⋯O and N—H⋯O hydrogen bonds (Table 1 ▸, Fig. 3 ▸), forming layers parallel to (100), defining 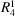 (6) and *C*(7) graph-set motifs (Bernstein *et al.*, 1995 ▸). Additional C—H⋯π inter­actions consolidate the layered arrangement (Table 1 ▸; Fig. 4 ▸). Between the layers, van der Waals inter­actions stabilize the packing, as revealed by Hirshfeld surface analysis.

## 4. Hirshfeld surface analysis

**Figure 5:**
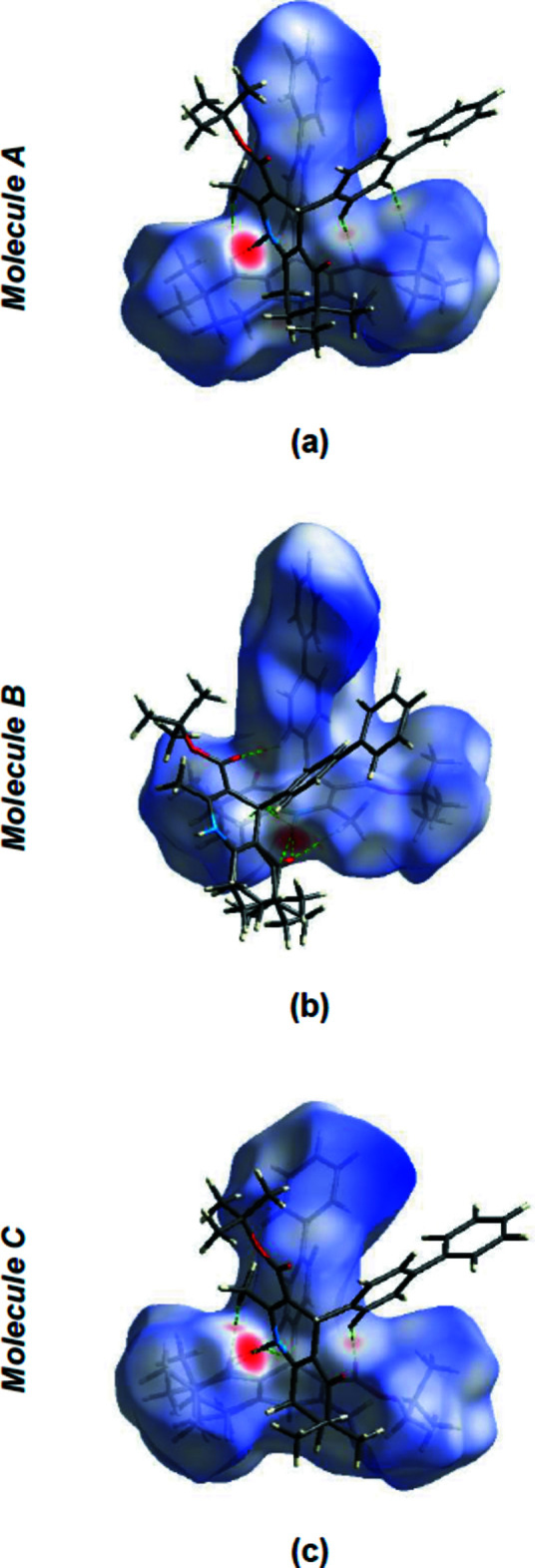
(*a*) View of the three-dimensional Hirshfeld surface for mol­ecule *A*; (*b*) view of the three-dimensional Hirshfeld surface for mol­ecule *B*; (*c*) view of the three-dimensional Hirshfeld surface for mol­ecule *C*. Some inter­molecular N—H⋯O and C—H⋯O inter­actions are shown as dashed lines.

*Crystal Explorer 17.5* (Spackman *et al.*, 2021 ▸) was used to construct Hirshfeld surfaces for the three independent mol­ecules; the disorder of mol­ecule *C* was included in the calculations. The *d*_norm_ mappings for mol­ecule *A* were performed in the range −0.5982 to +2.4710 a.u., for mol­ecule *B* in the range −0.6131 to +2.5190 a.u., and for mol­ecule *C* in the range −0.6097 to +2.4293 a.u.. On the *d*_norm_ surfaces, bright-red spots show the locations of N—H⋯O and C—H⋯O inter­actions (Fig. 5 ▸ *a* for mol­ecule *A*, Fig. 5 ▸ *b* for mol­ecule *B*, and Fig. 5 ▸ *c* for mol­ecule *C*).

**Figure 6:**
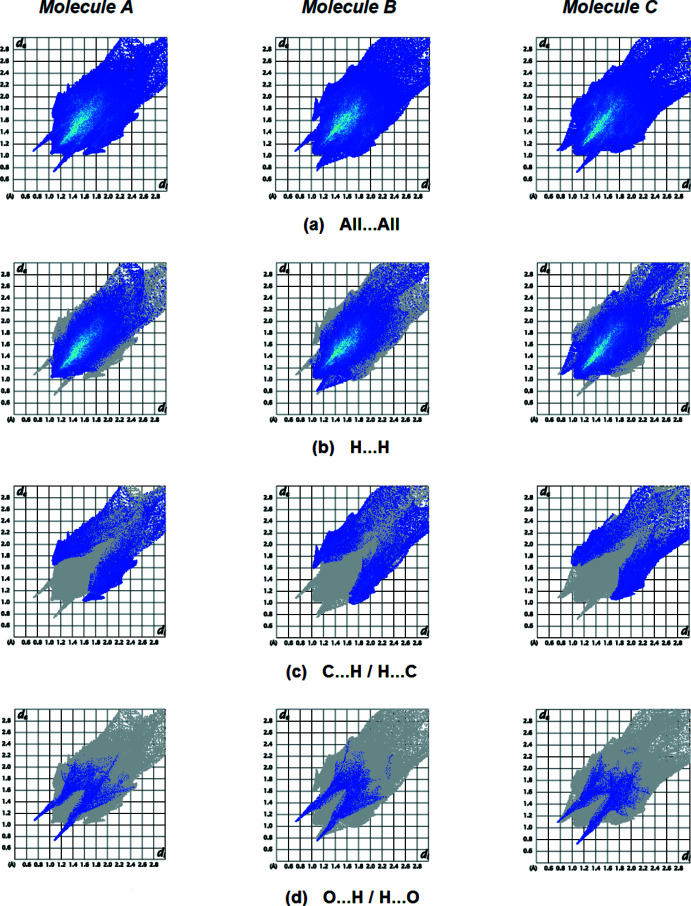
The two-dimensional fingerprint plots for mol­ecules *A*, *B* and *C* showing (*a*) all inter­actions, and delineated into (*b*) H⋯H, (*c*) C⋯H/H⋯C and (*d*) O⋯H/H⋯O inter­actions. The *d*_i_ and *d*_e_ values are the closest inter­nal and external distances (in Å) from given points on the Hirshfeld surface.

Fingerprint plots (Fig. 6 ▸) reveal that H⋯H inter­actions make the largest contributions (69.6% for mol­ecule *A*, 69.9% for mol­ecule *B*, and 70.1% for mol­ecule *C*) to the overall surface (Table 2 ▸). C⋯H/H⋯C (20.3% for *A*, 20.6% for *B*, and 20.3% for *C*) contacts are also significant. Table 3 ▸ lists the contributions of additional, less notable inter­actions. As seen in Table 3 ▸, the relevant contacts around mol­ecules *A*, *B*, and *C* are quite similar.

## 5. Database survey

A search of the Cambridge Structural Database (CSD, Version 5.42, update of September 2021; Groom *et al.*, 2016 ▸) for similar structures with the 1,4,5,6,7,8-hexa­hydro­quinoline unit revealed seven closely related entries: ethyl 4-(4-bromo­phen­yl)-2,7,7-trimethyl-5-oxo-1,4,5,6,7,8-hexa­hydro­quinoline-3-carboxyl­ate [CSD refcode LOQCAX (**I**); Steiger *et al.*, 2014 ▸], ethyl 4-(3-hy­droxy­phen­yl)-2,7,7-trimethyl-5-oxo-1,4,5,6,7,8-hexa­hydro­quinoline-3-carboxyl­ate [PUGCIE (**II**); Mookiah *et al.*, 2009 ▸], (*RR*,*SS*)-methyl 4-(2,4-chloro­phen­yl)-2,7-dimethyl-5-oxo-1,4,5,6,7,8-hexa­hydro­quinoline-3-carb­oxy­l­ate (*RS*,*SR*)-methyl 4-(2,4-chloro­phen­yl)-2,7-dimethyl-5-oxo-1,4,5,6,7,8-hexa­hydro­quinoline-3-carboxyl­ate [UCOLOO (**III**); Linden *et al.*, 2006 ▸], ethyl 2,7,7-trimethyl-4-(1-methyl-1*H*-indol-3-yl)-5-oxo-1,4,5,6,7,8-hexa­hydro­quinoline-3-carb­oxyl­ate [NEQMON (**IV**); Öztürk Yildirim *et al.*, 2013 ▸], (+/−)-methyl 4-(2,3-di­fluoro­phen­yl)-2,6,6-trimethyl-5-oxo-1,4,5,6,7,8-hexa­hydro­quinoline-3-carboxyl­ate [DAYJET (**V**); Linden *et al.*, 2005 ▸], benzyl 4-(3-chloro-2-fluoro­phen­yl)-2-methyl-5-oxo-4,5,6,7-tetra­hydro-1*H*-cyclo­penta­[*b*]pyridine-3-carboxyl­ate [IMEJOA (**VI**); Linden *et al.*, 2011 ▸], and ethyl 4-(5-bromo-1*H*-indol-3-yl)-2,6,6-trimethyl-5-oxo-1,4,5,6,7,8-hexa­hydro­quinoline-3-carboxyl­ate [PECPUK (**VII**); Gündüz *et al.*, 2012 ▸].

In (**I**), hydrogen bonds are formed between the N—H group of one mol­ecule and the carbonyl O atom in the cyclo­hexa­none ring of an adjacent mol­ecule. These hydrogen bonds link the mol­ecules into extended chains running along [001]. In the crystal of (**II**), mol­ecules are linked by N—H⋯O and O—H⋯O hydrogen bonds into layers parallel to (101). The network includes 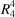 (30) and 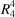 (34) graph-set motifs. In (**III**), an inter­molecular N—H⋯O hydrogen bond between the amine group and the carbonyl O atom of the cyclo­hexenone ring of a neighboring mol­ecule links the mol­ecules into extended chains parallel to [101]. These inter­actions can be described by graph-set motif *C*(6). In the crystal of (**IV**), N—H⋯O hydrogen bonds connect the mol­ecules into *C*(6) chains parallel to [010], and pairs of weak C—H⋯O hydrogen bonds link inversion-related chains into a ladder motif through 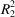 (18) rings. A weak intra­molecular C—H⋯O hydrogen bond is also observed. In (**V**), the crystal structure exhibits an inter­molecular N—H⋯O hydrogen-bonding inter­action involving the carbonyl O atom of the oxo­cyclo­hexene ring, whereby the mol­ecules are linked into *C*(6) chains parallel to [100]. In (**VI**), the frequently observed inter­molecular N—H⋯O hydrogen bond between the amine group and the carbonyl O atom of the oxo­cyclo­pentene ring of a neighboring mol­ecule links the mol­ecules into extended *C*(6) chains parallel to [010]; there are no other significant inter­molecular inter­actions. In the crystal of (**VII**), mol­ecules are linked by pairs of N—H⋯O hydrogen bonds, forming dimers with 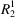 (6) ring motifs. These dimers are connected by N—H⋯O hydrogen bonds, generating chains along [110]. A C—H⋯O contact occurs between the independent mol­ecules.

## 6. Synthesis and crystallization

**Figure 7:**
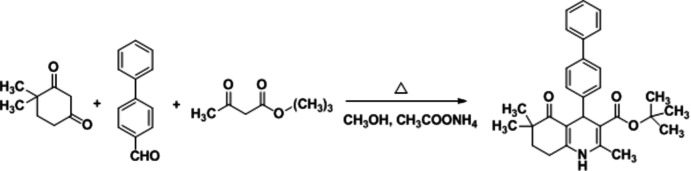
Schematic synthetic route for the title compound.

The title compound was synthesized *via* a Hantzsch reaction. 4,4-Di­methyl­cyclo­hexane-1,3-dione (1 mmol), [1,1′-biphen­yl]-4-carbaldehyde (1 mmol), *tert*-butyl aceto­acetate (1 mmol), and ammonium acetate (5 mmol) were refluxed for 8 h in absolute methanol (10 ml). The reaction mixture was monitored by TLC, and after completion of the reaction was cooled to room temperature. The obtained precipitate was filtered and recrystallized from ethanol for further purification. The synthetic route is shown in Fig. 7 ▸.

Yellowish solid, m.p. 520-522 K; yield: 41%. IR (ν, cm^−1^) 3284 (N—H, stretching), 3067 (C—H stretching, aromatic), 2966 (C—H stretching, aliphatic) 1671 (C=O stretching, ester), 1597 (C=O stretching, ketone). ^1^H NMR (DMSO-*d*_6_) δ: 0.88 (3H; *s*; 6-CH_3_), 0.97 (3H; *s*; 6-CH_3_), 1.32 [9H, *s*, C(CH_3_)_3_], 1.70–1.71 (2H; *m*; quinoline H7), 2.23 (3H; *s*; 2-CH_3_), 2.47–2.50 (2H; *m*; quinoline H8), 4.82 (1H; *s*; quinoline H4), 7.19–7.21 (2H, *m*, Ar-H), 7.27–7.31 (H, *m*, Ar-H), 7.38–7.48 (4H, *m*, Ar-H), 7.57–7.59 (2H, *m*, Ar-H), 8.98 (1H, *s*; NH). ^13^C NMR (DMSO-*d*_6_) δ: 18.2, 22.9, 24.1, 25.1, 27.9, 34.1, 36.0, 40.0, 78.7, 104.6, 108.8, 126.0, 126.3, 127.0, 127.9, 128.7, 137.3, 140.1, 143.8, 147.1, 149.8, 166.4, 199.3. Analysis calculated for C_29_H_33_NO_3_: C 78.52, H 7.5, N 3.16. Found: C 78.30, H 7.602, N 3.19.

## 7. Refinement details

Crystal data, data collection and structure refinement details are summarized in Table 4 ▸. All C-bound H atoms were positioned geometrically and allowed to ride on their parent atoms, with C—H = 0.95 Å for aryl-H atoms, C—H = 0.99 Å for methyl­ene groups, C—H = 1.00 Å for methine groups and C—H = 0.98 Å for methyl groups, with *U*_iso_(H) = 1.5*U*_eq_(C) for methyl groups and *U*_iso_(H) = 1.2*U*_eq_(C) for other hydrogen atoms. The H atoms of the NH groups were found in a difference-Fourier map and refined freely (see Table 1 ▸).

In mol­ecule *C*, except the fused carbon atoms (C4*C* and C9*C*) and the carbonyl oxygen atom (O1*C*) of the 6,6-di­methyl­cyclo­hex-2-en-1-one group (**C4** ***C***–C5*C*/C5*F*–C6*C*/C6*F*–C7*C*/C7*F*–C8*C*/C8*F*–**C9** ***C***–**O1** ***C***–C16*C*/C16*F*–C17*C*/C17*F*), the other C atoms are disordered over two sets of sites with a refined occupancy ratio of 0.716 (4):0.284 (4). For the disordered components, the EADP instruction was used in the final cycles of the refinement.

## Supplementary Material

Crystal structure: contains datablock(s) I. DOI: 10.1107/S2056989022007022/wm5652sup1.cif

Structure factors: contains datablock(s) I. DOI: 10.1107/S2056989022007022/wm5652Isup2.hkl

Supporting information file. DOI: 10.1107/S2056989022007022/wm5652Isup3.cml

CCDC reference: 2185278

Additional supporting information:  crystallographic information; 3D view; checkCIF report

## Figures and Tables

**Table 1:** Hydrogen-bond geometry (Å, °) *Cg*4, *Cg*8, *Cg*12 and *Cg*13 are the centroids of the C18*C*–C23*C*, C18*A*–C23*A*, C18*B*–C23*B*, and C18*B*–C29*B* rings, respectively.

*D*—H⋯*A*	*D*—H	H⋯*A*	*D*⋯*A*	*D*—H⋯*A*
N1*C*—H1*NC*⋯O1*B* ^i^	0.92 (5)	1.94 (5)	2.843 (4)	165 (5)
N1*B*—H1*NB*⋯O1*C*	0.95 (4)	1.88 (4)	2.811 (4)	168 (3)
N1*A*—H1*NA*⋯O1*A* ^ii^	0.93 (5)	1.92 (5)	2.842 (4)	174 (4)
C10*A*—H10*B*⋯O1*A* ^ii^	0.98	2.60	3.443 (5)	145
C10*B*—H10*F*⋯O1*C*	0.98	2.47	3.302 (5)	143
C13*A*—H13*A*⋯O2*A*	0.98	2.45	2.978 (7)	113
C13*B*—H13*D*⋯O2*B*	0.98	2.47	3.023 (6)	115
C15*A*—H15*A*⋯O2*A*	0.98	2.41	2.999 (7)	118
C15*B*—H15*D*⋯O2*B*	0.98	2.41	2.965 (6)	116
C15*C*—H15*G*⋯O2*C*	0.98	2.49	3.022 (5)	114
C23*A*—H23*A*⋯O2*A* ^ii^	0.95	2.57	3.403 (4)	147
C23*B*—H23*B*⋯O2*C*	0.95	2.55	3.389 (5)	147
C23*C*—H23*C*⋯O2*B* ^i^	0.95	2.60	3.407 (4)	144
C15*A*—H15*B*⋯*Cg*13	0.98	2.82	3.771 (6)	165
C27*A*—H27*A*⋯*Cg*4^ii^	0.95	2.75	3.578 (4)	146
C27*B*—H27*B*⋯*Cg*8	0.95	2.63	3.493 (5)	150
C27*C*—H27*C*⋯*Cg*12^iii^	0.95	2.84	3.632 (7)	142

Symmetry codes: (i) 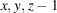 ; (ii) 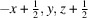 ; (iii) 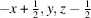 .

**Table 2:** Summary of short inter­atomic contacts (Å) in the title compound

Contact	Distance	Symmetry operation
O1*C*⋯H1*NB*	1.88	*x*, *y*, *z*
H1*NC*⋯O1*B*	1.94	*x*, *y*, −1 + *z*
H10*I*⋯H14*B*	2.40	1 − *x*, 1 − *y*, − 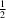 + *z*
C8*C*⋯H16*K*	3.07	1 − *x*, −*y*, − 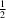 + *z*
H10*G*⋯H13*H*	2.43	1 − *x*, 1 − *y*, − 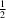 + *z*
C19*C*⋯H27*A*	2.86	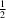 − *x*, *y*, − 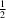 + *z*
H26*C*⋯C20*B*	2.90	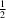 − *x*, *y*, − 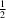 + *z*
H17*K*⋯H10*E*	2.07	1 − *x*, −*y*, − 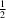 + *z*
H16*G*⋯H17*A*	2.52	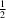 − *x*, −1 + *y*, − 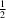 + *z*
H20*C*⋯H28*C*	2.56	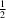 − *x*, *y*, − 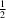 + *z*
O1*A*⋯H1*NA*	1.92	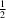 − *x*, *y*, − 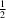 + *z*
H7*AA*⋯H15*E*	2.49	*x*, 1 + *y*, *z*
H10*B*⋯H17*D*	2.51	1 − *x*, 1 − *y*, 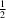 + *z*
H16*A*⋯H17*F*	2.47	− 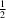 + *x*, 1 − *y*, *z*
C19*A*⋯H27*B*	2.89	*x*, *y*, *z*
H16*B*⋯C10*B*	2.97	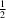 − *x*, *y*, − 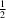 + *z*
H28*A*⋯H20*B*	2.42	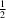 − *x*, *y*, − 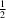 + *z*

**Table 3:** Percentage contributions of inter­atomic contacts to the Hirshfeld surfaces for the mol­ecules *A*, *B* and *C* of the title compound

Contact	% for *A*	% for *B*	% for *C*
H⋯H	69.6	69.9	70.1
C⋯H/H⋯C	20.3	20.6	20.3
O⋯H/H⋯O	8.6	8.6	8.4
N⋯H/H⋯N	1.1	0.8	0.9
C⋯C	0.5	0.1	0.4

**Table 4:** Experimental details

Crystal data	
Chemical formula	C_29_H_33_NO_3_
*M*_r_	443.56
Crystal system, space group	Orthorhombic, *P* *c* *a*2_1_
Temperature (K)	100
*a*, *b*, *c* (Å)	33.2247 (14), 19.0904 (7), 12.0370 (3)
*V* (Å^3^)	7634.7 (5)
*Z*	12
Radiation type	Mo *K*α
μ (mm^−1^)	0.07
Crystal size (mm)	0.29 × 0.17 × 0.04

Data collection	
Diffractometer	SuperNova, Dual, Cu at zero, Atlas
Absorption correction	Gaussian (*CrysAlis PRO*; Rigaku OD, 2015 ▸)
*T*_min_, *T*_max_	0.984, 0.997
No. of measured, independent and observed [*I* > 2σ(*I*)] reflections	69430, 26062, 13388
*R*_int_	0.091
(sin θ/λ)_max_ (Å^−1^)	0.815

Refinement	
*R*[*F* ^2^ > 2σ(*F* ^2^)], *wR*(*F* ^2^), *S*	0.077, 0.212, 1.02
No. of reflections	26062
No. of parameters	943
No. of restraints	13
H-atom treatment	H atoms treated by a mixture of independent and constrained refinement
Δρ_max_, Δρ_min_ (e Å^−3^)	0.58, −0.37

Computer programs: *CrysAlis PRO* (Rigaku OD, 2015 ▸), SHELXT (Sheldrick, 2015*a* ▸), *SHELXL* (Sheldrick, 2015*b* ▸), *ORTEP-3 for Windows* (Farrugia, 2012 ▸), and *PLATON* (Spek, 2020 ▸).

## supplementary crystallographic information

### Crystal data

**Table:**

C_29_H_33_NO_3_	*D*_x_ = 1.158 Mg m^−^^3^
*M**_r_* = 443.56	Mo *K*α radiation, λ = 0.71073 Å
Orthorhombic, *P**c**a*2_1_	Cell parameters from 9135 reflections
*a* = 33.2247 (14) Å	θ = 3.7–29.8°
*b* = 19.0904 (7) Å	µ = 0.07 mm^−^^1^
*c* = 12.0370 (3) Å	*T* = 100 K
*V* = 7634.7 (5) Å^3^	Plate, colorless
*Z* = 12	0.28 × 0.17 × 0.04 mm
*F*(000) = 2856	

### Data collection

**Table:**

SuperNova, Dual, Cu at zero, Atlas diffractometer	26062 independent reflections
Radiation source: micro-focus sealed X-ray tube	13388 reflections with *I* > 2σ(*I*)
Detector resolution: 10.6501 pixels mm^-1^	*R*_int_ = 0.091
ω scans	θ_max_ = 35.4°, θ_min_ = 3.4°
Absorption correction: gaussian (CrysAlisPro; Rigaku OD, 2015\bbr021)	*h* = −52→45
*T*_min_ = 0.984, *T*_max_ = 0.997	*k* = −30→18
69430 measured reflections	*l* = −12→19

### Refinement

**Table:**

Refinement on *F*^2^	13 restraints
Least-squares matrix: full	Hydrogen site location: mixed
*R*[*F*^2^ > 2σ(*F*^2^)] = 0.077	H atoms treated by a mixture of independent and constrained refinement
*wR*(*F*^2^) = 0.212	*w* = 1/[σ^2^(*F*_o_^2^) + (0.0858*P*)^2^ + 0.3289*P*] where *P* = (*F*_o_^2^ + 2*F*_c_^2^)/3
*S* = 1.02	(Δ/σ)_max_ < 0.001
26062 reflections	Δρ_max_ = 0.58 e Å^−^^3^
943 parameters	Δρ_min_ = −0.37 e Å^−^^3^

### Special details

**Table:**

Geometry. All esds (except the esd in the dihedral angle between two l.s. planes) are estimated using the full covariance matrix. The cell esds are taken into account individually in the estimation of esds in distances, angles and torsion angles; correlations between esds in cell parameters are only used when they are defined by crystal symmetry. An approximate (isotropic) treatment of cell esds is used for estimating esds involving l.s. planes.

### Fractional atomic coordinates and isotropic or equivalent isotropic displacement parameters (Å^2^)

**Table:**

	*x*	*y*	*z*	*U*_iso_*/*U*_eq_	Occ. (<1)
O1C	0.46360 (9)	0.08706 (13)	0.3497 (2)	0.0319 (6)	
O2C	0.45841 (11)	0.33966 (14)	0.4075 (2)	0.0401 (8)	
O3C	0.48150 (10)	0.41061 (13)	0.2723 (2)	0.0333 (7)	
N1C	0.48126 (9)	0.24320 (15)	0.0522 (2)	0.0210 (6)	
H1NC	0.4898 (17)	0.253 (3)	−0.019 (4)	0.054 (15)*	
C1C	0.47707 (11)	0.30241 (18)	0.1187 (3)	0.0217 (7)	
C2C	0.46516 (11)	0.29472 (18)	0.2253 (3)	0.0215 (7)	
C3C	0.44911 (11)	0.22483 (17)	0.2661 (3)	0.0195 (6)	
H3CA	0.457116	0.219390	0.345766	0.023*	
C4C	0.46876 (12)	0.16588 (18)	0.2003 (3)	0.0223 (7)	
C9C	0.48166 (11)	0.17716 (18)	0.0953 (3)	0.0216 (7)	
C10C	0.48761 (13)	0.36843 (19)	0.0585 (3)	0.0280 (8)	
H10G	0.469779	0.406270	0.083077	0.042*	
H10H	0.484358	0.361273	−0.021631	0.042*	
H10I	0.515606	0.381025	0.074573	0.042*	
C11C	0.46732 (13)	0.34996 (18)	0.3106 (3)	0.0252 (8)	
C12C	0.48964 (15)	0.4703 (2)	0.3458 (3)	0.0363 (10)	
C13C	0.52017 (17)	0.4500 (3)	0.4339 (4)	0.0531 (14)	
H13G	0.506416	0.426122	0.495179	0.080*	
H13H	0.533542	0.492149	0.461951	0.080*	
H13I	0.540253	0.418457	0.401344	0.080*	
C14C	0.5076 (2)	0.5231 (2)	0.2654 (4)	0.0633 (17)	
H14G	0.489180	0.529980	0.202794	0.095*	
H14H	0.533472	0.505606	0.237940	0.095*	
H14I	0.511733	0.567845	0.303760	0.095*	
C15C	0.45081 (16)	0.4972 (2)	0.3945 (4)	0.0455 (12)	
H15G	0.440597	0.463492	0.449029	0.068*	
H15H	0.430940	0.503272	0.335077	0.068*	
H15I	0.455634	0.542314	0.430975	0.068*	
C5C	0.4736 (4)	0.0987 (5)	0.2485 (11)	0.0182 (16)	0.716 (4)
C6C	0.4829 (2)	0.0353 (4)	0.1732 (5)	0.0231 (13)	0.716 (4)
C7C	0.51262 (18)	0.0589 (3)	0.0831 (4)	0.0254 (10)	0.716 (4)
H7CA	0.538459	0.071720	0.118645	0.030*	0.716 (4)
H7CB	0.517837	0.019137	0.032286	0.030*	0.716 (4)
C8C	0.4976 (7)	0.1206 (6)	0.0158 (11)	0.0219 (16)	0.716 (4)
H8CA	0.475884	0.105316	−0.034922	0.026*	0.716 (4)
H8CB	0.519848	0.139966	−0.029617	0.026*	0.716 (4)
C16C	0.44347 (19)	0.0096 (3)	0.1220 (5)	0.0347 (12)	0.716 (4)
H16G	0.425520	−0.006979	0.181001	0.052*	0.716 (4)
H16H	0.449072	−0.028783	0.070302	0.052*	0.716 (4)
H16I	0.430524	0.048234	0.081918	0.052*	0.716 (4)
C17C	0.5016 (2)	−0.0234 (3)	0.2432 (5)	0.0392 (14)	0.716 (4)
H17G	0.483035	−0.036371	0.303037	0.059*	0.716 (4)
H17H	0.527040	−0.007118	0.275277	0.059*	0.716 (4)
H17I	0.506661	−0.064298	0.195979	0.059*	0.716 (4)
C5F	0.4673 (10)	0.0923 (15)	0.258 (3)	0.0182 (16)	0.284 (4)
C6F	0.4902 (6)	0.0350 (11)	0.1928 (15)	0.0231 (13)	0.284 (4)
C7F	0.4842 (5)	0.0458 (6)	0.0679 (10)	0.0254 (10)	0.284 (4)
H7FA	0.500950	0.011719	0.026745	0.030*	0.284 (4)
H7FB	0.455677	0.036907	0.048734	0.030*	0.284 (4)
C8F	0.495 (2)	0.1197 (14)	0.032 (3)	0.0219 (16)	0.284 (4)
H8FA	0.525239	0.122302	0.030151	0.026*	0.284 (4)
H8FB	0.485832	0.126208	−0.044762	0.026*	0.284 (4)
C16F	0.4713 (5)	−0.0345 (7)	0.2282 (12)	0.0347 (12)	0.284 (4)
H16J	0.442582	−0.034222	0.210125	0.052*	0.284 (4)
H16K	0.474775	−0.040681	0.308485	0.052*	0.284 (4)
H16L	0.484577	−0.073023	0.188848	0.052*	0.284 (4)
C17F	0.5351 (5)	0.0358 (8)	0.2264 (11)	0.0392 (14)	0.284 (4)
H17J	0.545785	0.083378	0.218202	0.059*	0.284 (4)
H17K	0.550199	0.003752	0.178458	0.059*	0.284 (4)
H17L	0.537749	0.020880	0.303986	0.059*	0.284 (4)
C18C	0.40349 (11)	0.22258 (16)	0.2602 (3)	0.0205 (7)	
C19C	0.37983 (13)	0.23298 (19)	0.3550 (3)	0.0287 (8)	
H19C	0.392433	0.238312	0.425295	0.034*	
C20C	0.33815 (13)	0.2356 (2)	0.3474 (3)	0.0334 (9)	
H20C	0.322649	0.241551	0.413031	0.040*	
C21C	0.31872 (13)	0.22971 (19)	0.2458 (3)	0.0314 (8)	
C22C	0.34244 (13)	0.2192 (2)	0.1517 (3)	0.0307 (8)	
H22C	0.329790	0.214837	0.081296	0.037*	
C23C	0.38374 (12)	0.21487 (19)	0.1584 (3)	0.0255 (7)	
H23C	0.399005	0.206566	0.092969	0.031*	
C24C	0.27417 (14)	0.2351 (2)	0.2377 (4)	0.0413 (10)	
C25C	0.25248 (19)	0.2794 (3)	0.3068 (6)	0.0682 (17)	
H25C	0.266339	0.305737	0.361770	0.082*	
C26C	0.2112 (2)	0.2862 (4)	0.2975 (7)	0.082 (2)	
H26C	0.197001	0.316292	0.346652	0.098*	
C27C	0.19067 (19)	0.2494 (4)	0.2174 (6)	0.075 (2)	
H27C	0.162381	0.255019	0.210179	0.090*	
C28C	0.21126 (18)	0.2037 (3)	0.1465 (6)	0.0662 (17)	
H28C	0.197013	0.177839	0.091665	0.079*	
C29C	0.25298 (16)	0.1963 (3)	0.1569 (5)	0.0510 (12)	
H29C	0.267107	0.165141	0.109322	0.061*	
C1B	0.42485 (12)	0.13678 (18)	0.6165 (3)	0.0226 (7)	
O1B	0.52165 (8)	0.27146 (15)	0.8489 (2)	0.0301 (6)	
O2B	0.39953 (9)	0.13889 (15)	0.9060 (2)	0.0337 (7)	
O3B	0.36660 (9)	0.08654 (15)	0.7645 (2)	0.0352 (7)	
N1B	0.45666 (10)	0.16087 (16)	0.5509 (2)	0.0238 (6)	
H1NB	0.4580 (11)	0.1419 (18)	0.478 (3)	0.015 (9)*	
C2B	0.42144 (11)	0.15947 (18)	0.7226 (3)	0.0215 (7)	
C3B	0.44784 (11)	0.21896 (17)	0.7645 (3)	0.0192 (6)	
H3BA	0.455138	0.208552	0.843420	0.023*	
C4B	0.48650 (11)	0.22308 (17)	0.6980 (3)	0.0202 (7)	
C5B	0.52170 (11)	0.25356 (17)	0.7503 (3)	0.0214 (7)	
C6B	0.56081 (13)	0.2624 (2)	0.6833 (3)	0.0283 (8)	
C7B	0.55222 (14)	0.2632 (2)	0.5602 (3)	0.0353 (9)	
H7BA	0.578069	0.260824	0.519484	0.042*	
H7BB	0.539192	0.308243	0.540951	0.042*	
C8B	0.52497 (12)	0.2027 (2)	0.5206 (3)	0.0274 (8)	
H8BA	0.516517	0.211170	0.442931	0.033*	
H8BB	0.540204	0.158121	0.522827	0.033*	
C9B	0.48827 (12)	0.19693 (18)	0.5940 (3)	0.0217 (7)	
C10B	0.39848 (14)	0.0853 (2)	0.5569 (3)	0.0327 (9)	
H10D	0.370242	0.099190	0.565137	0.049*	
H10E	0.402363	0.038514	0.588696	0.049*	
H10F	0.405593	0.084498	0.477890	0.049*	
C11B	0.39505 (12)	0.12824 (19)	0.8071 (3)	0.0249 (7)	
C12B	0.34032 (15)	0.0438 (2)	0.8350 (4)	0.0408 (11)	
C13B	0.36627 (19)	−0.0084 (3)	0.8969 (5)	0.0636 (16)	
H13D	0.382559	0.016253	0.952372	0.095*	
H13E	0.384013	−0.032506	0.844298	0.095*	
H13F	0.349019	−0.042810	0.934182	0.095*	
C14B	0.31430 (18)	0.0074 (3)	0.7477 (4)	0.0605 (16)	
H14D	0.300055	0.042709	0.703607	0.091*	
H14E	0.294700	−0.023052	0.784723	0.091*	
H14F	0.331456	−0.020787	0.698874	0.091*	
C15B	0.31514 (17)	0.0884 (3)	0.9105 (4)	0.0538 (14)	
H15D	0.332446	0.110615	0.966285	0.081*	
H15E	0.295027	0.059134	0.947783	0.081*	
H15F	0.301516	0.124668	0.866833	0.081*	
C16B	0.58283 (17)	0.3280 (3)	0.7172 (5)	0.0588 (15)	
H16D	0.567336	0.369113	0.693858	0.088*	
H16E	0.609364	0.328881	0.681709	0.088*	
H16F	0.586101	0.328634	0.798126	0.088*	
C17B	0.58741 (17)	0.1989 (3)	0.7168 (4)	0.0548 (14)	
H17D	0.614125	0.203879	0.683244	0.082*	
H17E	0.574862	0.155539	0.690542	0.082*	
H17F	0.590032	0.197348	0.797901	0.082*	
C18B	0.42474 (11)	0.28796 (17)	0.7628 (3)	0.0201 (6)	
C19B	0.40710 (11)	0.31303 (19)	0.8600 (3)	0.0236 (7)	
H19B	0.411061	0.288512	0.927788	0.028*	
C20B	0.38374 (12)	0.37360 (19)	0.8589 (3)	0.0278 (8)	
H20B	0.372096	0.390038	0.926144	0.033*	
C21B	0.37724 (12)	0.41026 (19)	0.7609 (3)	0.0271 (8)	
C22B	0.39484 (12)	0.38486 (19)	0.6634 (3)	0.0270 (8)	
H22B	0.390634	0.408871	0.595268	0.032*	
C23B	0.41843 (12)	0.32481 (18)	0.6654 (3)	0.0243 (7)	
H23B	0.430470	0.308722	0.598470	0.029*	
C24B	0.35100 (13)	0.4735 (2)	0.7599 (3)	0.0306 (8)	
C25B	0.32067 (16)	0.4813 (3)	0.6810 (4)	0.0496 (12)	
H25B	0.317420	0.446661	0.624996	0.059*	
C26B	0.29521 (18)	0.5386 (3)	0.6827 (5)	0.0590 (15)	
H26B	0.274837	0.543358	0.627813	0.071*	
C27B	0.29953 (17)	0.5892 (2)	0.7652 (4)	0.0494 (12)	
H27B	0.281795	0.628257	0.767420	0.059*	
C28B	0.32936 (16)	0.5828 (2)	0.8431 (4)	0.0449 (11)	
H28B	0.332575	0.617497	0.899039	0.054*	
C29B	0.35499 (15)	0.5249 (2)	0.8398 (4)	0.0392 (10)	
H29B	0.375687	0.520736	0.893877	0.047*	
O1A	0.19045 (8)	0.90494 (14)	0.6522 (2)	0.0291 (6)	
O2A	0.31575 (10)	0.77814 (17)	0.5886 (2)	0.0413 (8)	
O3A	0.35749 (9)	0.75149 (15)	0.7299 (2)	0.0327 (6)	
N1A	0.27887 (10)	0.85463 (16)	0.9468 (2)	0.0225 (6)	
H1NA	0.2869 (14)	0.872 (2)	1.015 (4)	0.034 (12)*	
C1A	0.30583 (12)	0.81797 (19)	0.8791 (3)	0.0234 (7)	
C2A	0.29537 (11)	0.80338 (18)	0.7730 (3)	0.0217 (7)	
C3A	0.25278 (11)	0.81758 (17)	0.7316 (3)	0.0216 (7)	
H3AA	0.254555	0.833656	0.652717	0.026*	
C4A	0.23328 (11)	0.87495 (17)	0.7985 (3)	0.0201 (7)	
C5A	0.20096 (11)	0.91507 (18)	0.7502 (3)	0.0225 (7)	
C6A	0.17859 (12)	0.96972 (19)	0.8190 (3)	0.0248 (7)	
C7A	0.20552 (13)	0.9979 (2)	0.9126 (3)	0.0334 (9)	
H7AA	0.226618	1.028171	0.879871	0.040*	
H7AB	0.189038	1.027278	0.962894	0.040*	
C8A	0.22563 (12)	0.9401 (2)	0.9804 (3)	0.0258 (8)	
H8AA	0.245670	0.960979	1.031508	0.031*	
H8AB	0.205142	0.915549	1.025665	0.031*	
C9A	0.24611 (12)	0.88866 (17)	0.9046 (3)	0.0213 (7)	
C10A	0.34394 (12)	0.8005 (2)	0.9392 (3)	0.0280 (8)	
H10A	0.348828	0.749983	0.934835	0.042*	
H10B	0.341518	0.814513	1.017266	0.042*	
H10C	0.366455	0.825680	0.904907	0.042*	
C11A	0.32364 (12)	0.77620 (19)	0.6876 (3)	0.0244 (7)	
C12A	0.39117 (12)	0.7288 (2)	0.6574 (3)	0.0343 (9)	
C13A	0.4052 (2)	0.7882 (3)	0.5884 (6)	0.082 (2)	
H13A	0.385439	0.797594	0.529805	0.123*	
H13B	0.431160	0.776395	0.554694	0.123*	
H13C	0.408238	0.829931	0.635093	0.123*	
C14A	0.42246 (17)	0.7077 (5)	0.7437 (5)	0.086 (2)	
H14A	0.434532	0.749834	0.776152	0.128*	
H14B	0.443469	0.679505	0.708135	0.128*	
H14C	0.409493	0.680156	0.802371	0.128*	
C15A	0.37950 (17)	0.6669 (3)	0.5897 (5)	0.0580 (15)	
H15A	0.357515	0.679747	0.539431	0.087*	
H15B	0.370586	0.629061	0.638992	0.087*	
H15C	0.402698	0.651017	0.546218	0.087*	
C16A	0.14186 (14)	0.9325 (2)	0.8690 (4)	0.0414 (11)	
H16A	0.123619	0.918108	0.809204	0.062*	
H16B	0.127771	0.964496	0.919351	0.062*	
H16C	0.150750	0.891041	0.910324	0.062*	
C17A	0.16505 (16)	1.0306 (2)	0.7470 (4)	0.0450 (11)	
H17A	0.149466	1.012812	0.683909	0.068*	
H17B	0.188688	1.055973	0.719545	0.068*	
H17C	0.148257	1.062366	0.791092	0.068*	
C18A	0.22898 (11)	0.74917 (18)	0.7339 (3)	0.0211 (7)	
C19A	0.22517 (12)	0.70862 (19)	0.6374 (3)	0.0255 (8)	
H19A	0.235632	0.725514	0.569005	0.031*	
C20A	0.20609 (13)	0.64357 (19)	0.6413 (3)	0.0281 (8)	
H20A	0.203371	0.617012	0.575007	0.034*	
C21A	0.19106 (12)	0.61693 (18)	0.7399 (3)	0.0262 (7)	
C22A	0.19480 (12)	0.6574 (2)	0.8352 (3)	0.0296 (8)	
H22A	0.184586	0.640152	0.903634	0.035*	
C23A	0.21326 (12)	0.7228 (2)	0.8321 (3)	0.0262 (8)	
H23A	0.215120	0.749805	0.898261	0.031*	
C24A	0.17181 (13)	0.5460 (2)	0.7452 (3)	0.0318 (9)	
C25A	0.17811 (16)	0.5014 (2)	0.8345 (4)	0.0472 (12)	
H25A	0.194331	0.516414	0.895040	0.057*	
C26A	0.16085 (19)	0.4349 (3)	0.8364 (5)	0.0583 (15)	
H26A	0.165798	0.404538	0.897385	0.070*	
C27A	0.13681 (17)	0.4132 (2)	0.7507 (5)	0.0480 (12)	
H27A	0.125111	0.367771	0.752173	0.058*	
C28A	0.12964 (17)	0.4572 (2)	0.6627 (4)	0.0490 (13)	
H28A	0.112501	0.442335	0.604053	0.059*	
C29A	0.14713 (15)	0.5229 (2)	0.6584 (4)	0.0415 (11)	
H29A	0.142360	0.552467	0.596335	0.050*	

### Atomic displacement parameters (Å^2^)

**Table:**

	*U*^11^	*U*^22^	*U*^33^	*U*^12^	*U*^13^	*U*^23^
O1C	0.051 (2)	0.0247 (13)	0.0196 (12)	0.0041 (12)	0.0034 (12)	0.0022 (10)
O2C	0.069 (2)	0.0285 (14)	0.0226 (13)	−0.0081 (15)	0.0078 (13)	−0.0045 (11)
O3C	0.052 (2)	0.0240 (13)	0.0240 (13)	−0.0068 (12)	0.0055 (12)	−0.0046 (10)
N1C	0.0224 (16)	0.0256 (14)	0.0149 (12)	−0.0024 (12)	0.0018 (11)	−0.0009 (11)
C1C	0.0212 (19)	0.0229 (16)	0.0210 (15)	0.0036 (14)	−0.0036 (12)	0.0010 (12)
C2C	0.0246 (19)	0.0239 (16)	0.0161 (14)	0.0031 (14)	−0.0019 (12)	−0.0019 (12)
C3C	0.0234 (18)	0.0215 (15)	0.0136 (13)	0.0037 (13)	−0.0006 (12)	−0.0008 (12)
C4C	0.023 (2)	0.0247 (16)	0.0187 (15)	0.0021 (14)	−0.0022 (12)	−0.0013 (12)
C9C	0.0209 (19)	0.0270 (17)	0.0168 (14)	0.0012 (14)	−0.0027 (12)	−0.0021 (12)
C10C	0.035 (2)	0.0270 (17)	0.0223 (16)	−0.0047 (16)	0.0009 (15)	0.0011 (14)
C11C	0.034 (2)	0.0207 (16)	0.0205 (16)	0.0028 (15)	0.0010 (14)	−0.0016 (12)
C12C	0.053 (3)	0.0259 (19)	0.0297 (19)	−0.0027 (19)	0.0014 (19)	−0.0097 (16)
C13C	0.053 (4)	0.045 (3)	0.061 (3)	0.006 (2)	−0.017 (3)	−0.021 (2)
C14C	0.110 (5)	0.035 (2)	0.045 (3)	−0.030 (3)	0.015 (3)	−0.012 (2)
C15C	0.051 (3)	0.034 (2)	0.052 (3)	0.000 (2)	−0.004 (2)	−0.017 (2)
C5C	0.017 (5)	0.021 (3)	0.017 (3)	−0.002 (2)	0.005 (2)	0.001 (2)
C6C	0.028 (4)	0.0228 (17)	0.019 (3)	0.004 (2)	−0.002 (2)	−0.002 (2)
C7C	0.029 (3)	0.022 (2)	0.026 (2)	0.003 (2)	0.001 (2)	−0.0031 (17)
C8C	0.028 (4)	0.0290 (18)	0.009 (5)	0.0022 (17)	0.005 (4)	−0.0020 (19)
C16C	0.042 (4)	0.030 (3)	0.032 (3)	0.001 (2)	0.002 (2)	−0.009 (2)
C17C	0.058 (4)	0.033 (3)	0.027 (3)	0.020 (3)	−0.002 (2)	−0.003 (2)
C5F	0.017 (5)	0.021 (3)	0.017 (3)	−0.002 (2)	0.005 (2)	0.001 (2)
C6F	0.028 (4)	0.0228 (17)	0.019 (3)	0.004 (2)	−0.002 (2)	−0.002 (2)
C7F	0.029 (3)	0.022 (2)	0.026 (2)	0.003 (2)	0.001 (2)	−0.0031 (17)
C8F	0.028 (4)	0.0290 (18)	0.009 (5)	0.0022 (17)	0.005 (4)	−0.0020 (19)
C16F	0.042 (4)	0.030 (3)	0.032 (3)	0.001 (2)	0.002 (2)	−0.009 (2)
C17F	0.058 (4)	0.033 (3)	0.027 (3)	0.020 (3)	−0.002 (2)	−0.003 (2)
C18C	0.0265 (19)	0.0162 (13)	0.0189 (14)	0.0028 (13)	0.0046 (13)	0.0023 (12)
C19C	0.033 (2)	0.0301 (19)	0.0229 (17)	0.0065 (17)	0.0063 (15)	0.0023 (14)
C20C	0.031 (2)	0.037 (2)	0.032 (2)	0.0077 (18)	0.0126 (17)	0.0007 (17)
C21C	0.026 (2)	0.0271 (18)	0.041 (2)	0.0035 (16)	0.0062 (17)	0.0057 (16)
C22C	0.024 (2)	0.035 (2)	0.032 (2)	−0.0013 (17)	−0.0020 (16)	0.0040 (16)
C23C	0.025 (2)	0.0289 (18)	0.0231 (16)	−0.0002 (15)	0.0003 (14)	0.0007 (14)
C24C	0.026 (2)	0.044 (2)	0.054 (3)	0.0030 (19)	0.0069 (19)	0.012 (2)
C25C	0.034 (3)	0.071 (4)	0.099 (5)	0.014 (3)	0.014 (3)	−0.007 (3)
C26C	0.035 (4)	0.084 (5)	0.126 (6)	0.016 (3)	0.021 (4)	0.003 (4)
C27C	0.024 (3)	0.084 (4)	0.116 (6)	0.011 (3)	0.006 (3)	0.031 (4)
C28C	0.035 (3)	0.082 (4)	0.082 (4)	−0.003 (3)	−0.005 (3)	0.031 (3)
C29C	0.031 (3)	0.059 (3)	0.063 (3)	−0.003 (2)	−0.001 (2)	0.017 (3)
C1B	0.026 (2)	0.0222 (16)	0.0201 (15)	0.0013 (14)	−0.0041 (13)	0.0027 (12)
O1B	0.0243 (15)	0.0471 (16)	0.0189 (12)	−0.0089 (12)	0.0020 (10)	−0.0034 (11)
O2B	0.0331 (18)	0.0481 (17)	0.0200 (12)	−0.0136 (13)	0.0004 (11)	−0.0024 (11)
O3B	0.0350 (18)	0.0450 (16)	0.0257 (13)	−0.0183 (13)	0.0066 (12)	−0.0055 (12)
N1B	0.0297 (18)	0.0278 (15)	0.0139 (12)	0.0022 (13)	−0.0001 (11)	−0.0024 (11)
C2B	0.0207 (18)	0.0262 (16)	0.0176 (15)	0.0014 (14)	−0.0003 (12)	0.0002 (12)
C3B	0.0215 (17)	0.0222 (15)	0.0140 (13)	−0.0005 (13)	−0.0007 (12)	0.0008 (12)
C4B	0.0200 (18)	0.0220 (16)	0.0187 (15)	0.0037 (13)	0.0012 (12)	0.0027 (12)
C5B	0.0205 (18)	0.0240 (16)	0.0198 (16)	0.0010 (13)	0.0025 (12)	0.0043 (12)
C6B	0.030 (2)	0.0313 (19)	0.0232 (17)	−0.0034 (17)	0.0063 (14)	−0.0010 (14)
C7B	0.032 (2)	0.042 (2)	0.031 (2)	−0.0033 (19)	0.0056 (17)	0.0034 (17)
C8B	0.025 (2)	0.038 (2)	0.0189 (15)	0.0042 (17)	0.0052 (13)	−0.0001 (14)
C9B	0.026 (2)	0.0226 (16)	0.0160 (14)	0.0043 (14)	0.0009 (13)	0.0024 (12)
C10B	0.035 (2)	0.036 (2)	0.0268 (18)	−0.0076 (18)	−0.0038 (16)	−0.0062 (16)
C11B	0.022 (2)	0.0288 (18)	0.0237 (16)	−0.0002 (15)	0.0001 (13)	−0.0028 (13)
C12B	0.044 (3)	0.043 (2)	0.035 (2)	−0.025 (2)	0.0019 (19)	0.0035 (18)
C13B	0.065 (4)	0.048 (3)	0.078 (4)	−0.016 (3)	0.009 (3)	0.020 (3)
C14B	0.059 (4)	0.073 (3)	0.050 (3)	−0.044 (3)	0.014 (2)	−0.018 (3)
C15B	0.044 (3)	0.072 (3)	0.046 (3)	−0.023 (3)	0.020 (2)	−0.005 (2)
C16B	0.038 (3)	0.082 (4)	0.056 (3)	−0.022 (3)	0.014 (2)	−0.004 (3)
C17B	0.045 (3)	0.083 (4)	0.036 (3)	0.020 (3)	0.002 (2)	0.009 (2)
C18B	0.0179 (17)	0.0232 (15)	0.0190 (14)	0.0008 (13)	−0.0030 (12)	−0.0010 (12)
C19B	0.023 (2)	0.0314 (18)	0.0169 (15)	0.0025 (15)	−0.0006 (12)	−0.0004 (13)
C20B	0.026 (2)	0.0313 (19)	0.0254 (17)	0.0046 (16)	0.0028 (14)	−0.0054 (14)
C21B	0.0221 (19)	0.0273 (18)	0.0318 (18)	0.0031 (14)	0.0003 (15)	0.0012 (15)
C22B	0.027 (2)	0.0312 (19)	0.0225 (16)	0.0056 (16)	−0.0022 (14)	0.0012 (14)
C23B	0.026 (2)	0.0274 (17)	0.0193 (16)	0.0041 (15)	0.0021 (13)	−0.0009 (13)
C24B	0.029 (2)	0.0309 (19)	0.0322 (19)	0.0077 (16)	0.0048 (16)	0.0023 (16)
C25B	0.040 (3)	0.048 (3)	0.061 (3)	0.011 (2)	−0.007 (2)	−0.001 (2)
C26B	0.040 (3)	0.056 (3)	0.081 (4)	0.021 (3)	−0.009 (3)	0.001 (3)
C27B	0.043 (3)	0.043 (2)	0.063 (3)	0.017 (2)	0.008 (2)	0.001 (2)
C28B	0.052 (3)	0.035 (2)	0.047 (3)	0.008 (2)	0.015 (2)	−0.0005 (19)
C29B	0.040 (3)	0.035 (2)	0.043 (2)	0.0089 (19)	0.009 (2)	0.0032 (18)
O1A	0.0263 (15)	0.0419 (15)	0.0191 (12)	0.0097 (12)	−0.0028 (10)	−0.0048 (11)
O2A	0.0344 (18)	0.071 (2)	0.0184 (13)	0.0237 (16)	0.0006 (11)	−0.0042 (13)
O3A	0.0216 (15)	0.0526 (17)	0.0237 (13)	0.0112 (13)	0.0013 (10)	−0.0044 (12)
N1A	0.0264 (17)	0.0281 (15)	0.0130 (12)	−0.0003 (13)	−0.0024 (11)	−0.0015 (11)
C1A	0.024 (2)	0.0279 (17)	0.0187 (15)	−0.0025 (15)	−0.0002 (12)	0.0023 (12)
C2A	0.0217 (19)	0.0252 (16)	0.0181 (15)	0.0023 (14)	0.0000 (12)	−0.0027 (13)
C3A	0.0218 (18)	0.0272 (17)	0.0158 (13)	0.0028 (14)	0.0011 (12)	−0.0007 (12)
C4A	0.0225 (19)	0.0212 (15)	0.0167 (14)	0.0018 (14)	0.0018 (12)	−0.0013 (12)
C5A	0.0261 (19)	0.0263 (16)	0.0150 (15)	0.0004 (14)	0.0015 (13)	−0.0008 (12)
C6A	0.025 (2)	0.0272 (17)	0.0223 (16)	0.0063 (15)	0.0007 (13)	−0.0014 (13)
C7A	0.031 (2)	0.037 (2)	0.0323 (19)	0.0028 (18)	0.0019 (16)	−0.0064 (16)
C8A	0.026 (2)	0.0319 (19)	0.0198 (15)	0.0006 (16)	0.0022 (13)	−0.0072 (14)
C9A	0.0247 (19)	0.0229 (15)	0.0162 (14)	−0.0021 (14)	0.0027 (12)	−0.0017 (12)
C10A	0.023 (2)	0.040 (2)	0.0214 (16)	0.0038 (16)	−0.0018 (14)	−0.0011 (15)
C11A	0.0205 (19)	0.0312 (18)	0.0217 (16)	0.0027 (15)	−0.0003 (13)	−0.0020 (13)
C12A	0.0164 (19)	0.058 (3)	0.0289 (19)	0.0097 (18)	0.0023 (14)	−0.0045 (18)
C13A	0.058 (4)	0.076 (4)	0.111 (5)	0.008 (3)	0.055 (4)	0.021 (4)
C14A	0.030 (3)	0.178 (7)	0.049 (3)	0.045 (4)	−0.011 (2)	−0.028 (4)
C15A	0.035 (3)	0.076 (4)	0.063 (3)	0.013 (3)	0.010 (2)	−0.025 (3)
C16A	0.036 (3)	0.046 (2)	0.043 (2)	0.000 (2)	0.0089 (19)	−0.0111 (19)
C17A	0.052 (3)	0.039 (2)	0.044 (3)	0.014 (2)	−0.002 (2)	−0.003 (2)
C18A	0.0194 (18)	0.0256 (16)	0.0184 (15)	0.0031 (14)	−0.0036 (12)	−0.0006 (12)
C19A	0.029 (2)	0.0271 (17)	0.0209 (16)	0.0026 (15)	−0.0019 (14)	−0.0017 (13)
C20A	0.033 (2)	0.0278 (18)	0.0240 (17)	0.0047 (16)	−0.0043 (15)	−0.0067 (14)
C21A	0.024 (2)	0.0237 (16)	0.0306 (19)	0.0052 (14)	−0.0006 (14)	−0.0020 (14)
C22A	0.029 (2)	0.035 (2)	0.0247 (18)	−0.0005 (17)	0.0003 (15)	0.0006 (15)
C23A	0.027 (2)	0.0324 (19)	0.0188 (16)	0.0003 (16)	−0.0011 (13)	−0.0038 (13)
C24A	0.029 (2)	0.0309 (19)	0.035 (2)	0.0008 (16)	0.0068 (16)	−0.0031 (16)
C25A	0.048 (3)	0.037 (2)	0.056 (3)	−0.007 (2)	−0.003 (2)	0.005 (2)
C26A	0.068 (4)	0.035 (2)	0.072 (4)	−0.005 (3)	0.010 (3)	0.012 (2)
C27A	0.050 (3)	0.033 (2)	0.061 (3)	−0.008 (2)	0.012 (2)	−0.006 (2)
C28A	0.049 (3)	0.041 (3)	0.057 (3)	−0.011 (2)	0.013 (2)	−0.016 (2)
C29A	0.044 (3)	0.035 (2)	0.045 (2)	−0.006 (2)	0.006 (2)	−0.0079 (19)

### Geometric parameters (Å, º)

**Table:**

O1C—C5F	1.11 (4)	C10B—H10D	0.9800
O1C—C5C	1.282 (12)	C10B—H10E	0.9800
O2C—C11C	1.219 (4)	C10B—H10F	0.9800
O3C—C11C	1.332 (4)	C12B—C15B	1.501 (7)
O3C—C12C	1.468 (4)	C12B—C13B	1.514 (7)
N1C—C9C	1.363 (4)	C12B—C14B	1.528 (6)
N1C—C1C	1.392 (4)	C13B—H13D	0.9800
N1C—H1NC	0.92 (5)	C13B—H13E	0.9800
C1C—C2C	1.351 (5)	C13B—H13F	0.9800
C1C—C10C	1.495 (5)	C14B—H14D	0.9800
C2C—C11C	1.474 (5)	C14B—H14E	0.9800
C2C—C3C	1.519 (5)	C14B—H14F	0.9800
C3C—C18C	1.518 (5)	C15B—H15D	0.9800
C3C—C4C	1.523 (5)	C15B—H15E	0.9800
C3C—H3CA	1.0000	C15B—H15F	0.9800
C4C—C9C	1.352 (5)	C16B—H16D	0.9800
C4C—C5C	1.416 (13)	C16B—H16E	0.9800
C4C—C5F	1.57 (3)	C16B—H16F	0.9800
C9C—C8F	1.41 (4)	C17B—H17D	0.9800
C9C—C8C	1.537 (15)	C17B—H17E	0.9800
C10C—H10G	0.9800	C17B—H17F	0.9800
C10C—H10H	0.9800	C18B—C23B	1.384 (5)
C10C—H10I	0.9800	C18B—C19B	1.393 (5)
C12C—C15C	1.507 (6)	C19B—C20B	1.393 (5)
C12C—C13C	1.518 (7)	C19B—H19B	0.9500
C12C—C14C	1.519 (6)	C20B—C21B	1.389 (5)
C13C—H13G	0.9800	C20B—H20B	0.9500
C13C—H13H	0.9800	C21B—C22B	1.398 (5)
C13C—H13I	0.9800	C21B—C24B	1.488 (5)
C14C—H14G	0.9800	C22B—C23B	1.389 (5)
C14C—H14H	0.9800	C22B—H22B	0.9500
C14C—H14I	0.9800	C23B—H23B	0.9500
C15C—H15G	0.9800	C24B—C29B	1.381 (6)
C15C—H15H	0.9800	C24B—C25B	1.393 (6)
C15C—H15I	0.9800	C25B—C26B	1.383 (7)
C5C—C6C	1.543 (8)	C25B—H25B	0.9500
C6C—C16C	1.529 (8)	C26B—C27B	1.394 (7)
C6C—C17C	1.534 (8)	C26B—H26B	0.9500
C6C—C7C	1.534 (9)	C27B—C28B	1.370 (7)
C7C—C8C	1.514 (13)	C27B—H27B	0.9500
C7C—H7CA	0.9900	C28B—C29B	1.396 (6)
C7C—H7CB	0.9900	C28B—H28B	0.9500
C8C—H8CA	0.9900	C29B—H29B	0.9500
C8C—H8CB	0.9900	O1A—C5A	1.246 (4)
C16C—H16G	0.9800	O2A—C11A	1.221 (4)
C16C—H16H	0.9800	O3A—C11A	1.322 (5)
C16C—H16I	0.9800	O3A—C12A	1.483 (5)
C17C—H17G	0.9800	N1A—C9A	1.365 (5)
C17C—H17H	0.9800	N1A—C1A	1.398 (5)
C17C—H17I	0.9800	N1A—H1NA	0.92 (4)
C5F—C6F	1.548 (18)	C1A—C2A	1.353 (5)
C6F—C16F	1.526 (19)	C1A—C10A	1.496 (5)
C6F—C7F	1.530 (17)	C2A—C11A	1.486 (5)
C6F—C17F	1.547 (19)	C2A—C3A	1.525 (5)
C7F—C8F	1.52 (2)	C3A—C4A	1.506 (5)
C7F—H7FA	0.9900	C3A—C18A	1.527 (5)
C7F—H7FB	0.9900	C3A—H3AA	1.0000
C8F—H8FA	0.9900	C4A—C9A	1.372 (5)
C8F—H8FB	0.9900	C4A—C5A	1.442 (5)
C16F—H16J	0.9800	C5A—C6A	1.525 (5)
C16F—H16K	0.9800	C6A—C17A	1.518 (6)
C16F—H16L	0.9800	C6A—C16A	1.535 (6)
C17F—H17J	0.9800	C6A—C7A	1.537 (5)
C17F—H17K	0.9800	C7A—C8A	1.527 (6)
C17F—H17L	0.9800	C7A—H7AA	0.9900
C18C—C23C	1.398 (5)	C7A—H7AB	0.9900
C18C—C19C	1.399 (5)	C8A—C9A	1.503 (5)
C19C—C20C	1.389 (6)	C8A—H8AA	0.9900
C19C—H19C	0.9500	C8A—H8AB	0.9900
C20C—C21C	1.387 (6)	C10A—H10A	0.9800
C20C—H20C	0.9500	C10A—H10B	0.9800
C21C—C22C	1.394 (6)	C10A—H10C	0.9800
C21C—C24C	1.487 (6)	C12A—C13A	1.480 (7)
C22C—C23C	1.377 (6)	C12A—C15A	1.488 (7)
C22C—H22C	0.9500	C12A—C14A	1.524 (7)
C23C—H23C	0.9500	C13A—H13A	0.9800
C24C—C25C	1.387 (7)	C13A—H13B	0.9800
C24C—C29C	1.411 (7)	C13A—H13C	0.9800
C25C—C26C	1.382 (9)	C14A—H14A	0.9800
C25C—H25C	0.9500	C14A—H14B	0.9800
C26C—C27C	1.374 (10)	C14A—H14C	0.9800
C26C—H26C	0.9500	C15A—H15A	0.9800
C27C—C28C	1.400 (9)	C15A—H15B	0.9800
C27C—H27C	0.9500	C15A—H15C	0.9800
C28C—C29C	1.399 (8)	C16A—H16A	0.9800
C28C—H28C	0.9500	C16A—H16B	0.9800
C29C—H29C	0.9500	C16A—H16C	0.9800
C1B—C2B	1.354 (5)	C17A—H17A	0.9800
C1B—N1B	1.397 (5)	C17A—H17B	0.9800
C1B—C10B	1.500 (5)	C17A—H17C	0.9800
O1B—C5B	1.235 (4)	C18A—C23A	1.387 (5)
O2B—C11B	1.216 (4)	C18A—C19A	1.402 (5)
O3B—C11B	1.338 (5)	C19A—C20A	1.395 (5)
O3B—C12B	1.466 (5)	C19A—H19A	0.9500
N1B—C9B	1.358 (5)	C20A—C21A	1.384 (5)
N1B—H1NB	0.95 (4)	C20A—H20A	0.9500
C2B—C11B	1.469 (5)	C21A—C22A	1.389 (5)
C2B—C3B	1.521 (5)	C21A—C24A	1.499 (5)
C3B—C4B	1.515 (5)	C22A—C23A	1.392 (5)
C3B—C18B	1.525 (5)	C22A—H22A	0.9500
C3B—H3BA	1.0000	C23A—H23A	0.9500
C4B—C9B	1.350 (5)	C24A—C25A	1.387 (6)
C4B—C5B	1.450 (5)	C24A—C29A	1.399 (6)
C5B—C6B	1.538 (5)	C25A—C26A	1.393 (7)
C6B—C16B	1.507 (7)	C25A—H25A	0.9500
C6B—C7B	1.510 (5)	C26A—C27A	1.369 (8)
C6B—C17B	1.553 (7)	C26A—H26A	0.9500
C7B—C8B	1.543 (6)	C27A—C28A	1.373 (7)
C7B—H7BA	0.9900	C27A—H27A	0.9500
C7B—H7BB	0.9900	C28A—C29A	1.384 (6)
C8B—C9B	1.509 (5)	C28A—H28A	0.9500
C8B—H8BA	0.9900	C29A—H29A	0.9500
C8B—H8BB	0.9900		
			
C11C—O3C—C12C	122.1 (3)	C1B—C10B—H10E	109.5
C9C—N1C—C1C	122.2 (3)	H10D—C10B—H10E	109.5
C9C—N1C—H1NC	123 (3)	C1B—C10B—H10F	109.5
C1C—N1C—H1NC	113 (3)	H10D—C10B—H10F	109.5
C2C—C1C—N1C	119.1 (3)	H10E—C10B—H10F	109.5
C2C—C1C—C10C	128.3 (3)	O2B—C11B—O3B	124.1 (4)
N1C—C1C—C10C	112.5 (3)	O2B—C11B—C2B	122.4 (4)
C1C—C2C—C11C	124.7 (3)	O3B—C11B—C2B	113.4 (3)
C1C—C2C—C3C	120.4 (3)	O3B—C12B—C15B	111.5 (4)
C11C—C2C—C3C	114.9 (3)	O3B—C12B—C13B	108.2 (4)
C18C—C3C—C2C	111.1 (3)	C15B—C12B—C13B	113.1 (4)
C18C—C3C—C4C	112.5 (3)	O3B—C12B—C14B	101.1 (3)
C2C—C3C—C4C	109.3 (3)	C15B—C12B—C14B	111.1 (4)
C18C—C3C—H3CA	107.9	C13B—C12B—C14B	111.2 (4)
C2C—C3C—H3CA	107.9	C12B—C13B—H13D	109.5
C4C—C3C—H3CA	107.9	C12B—C13B—H13E	109.5
C9C—C4C—C5C	119.4 (5)	H13D—C13B—H13E	109.5
C9C—C4C—C3C	120.3 (3)	C12B—C13B—H13F	109.5
C5C—C4C—C3C	120.3 (4)	H13D—C13B—H13F	109.5
C9C—C4C—C5F	124.6 (9)	H13E—C13B—H13F	109.5
C3C—C4C—C5F	114.6 (9)	C12B—C14B—H14D	109.5
C4C—C9C—N1C	120.0 (3)	C12B—C14B—H14E	109.5
C4C—C9C—C8F	118.8 (10)	H14D—C14B—H14E	109.5
N1C—C9C—C8F	121.2 (10)	C12B—C14B—H14F	109.5
C4C—C9C—C8C	125.4 (4)	H14D—C14B—H14F	109.5
N1C—C9C—C8C	114.5 (4)	H14E—C14B—H14F	109.5
C1C—C10C—H10G	109.5	C12B—C15B—H15D	109.5
C1C—C10C—H10H	109.5	C12B—C15B—H15E	109.5
H10G—C10C—H10H	109.5	H15D—C15B—H15E	109.5
C1C—C10C—H10I	109.5	C12B—C15B—H15F	109.5
H10G—C10C—H10I	109.5	H15D—C15B—H15F	109.5
H10H—C10C—H10I	109.5	H15E—C15B—H15F	109.5
O2C—C11C—O3C	123.9 (3)	C6B—C16B—H16D	109.5
O2C—C11C—C2C	122.6 (3)	C6B—C16B—H16E	109.5
O3C—C11C—C2C	113.5 (3)	H16D—C16B—H16E	109.5
O3C—C12C—C15C	109.9 (4)	C6B—C16B—H16F	109.5
O3C—C12C—C13C	110.2 (3)	H16D—C16B—H16F	109.5
C15C—C12C—C13C	112.8 (4)	H16E—C16B—H16F	109.5
O3C—C12C—C14C	101.7 (3)	C6B—C17B—H17D	109.5
C15C—C12C—C14C	111.0 (4)	C6B—C17B—H17E	109.5
C13C—C12C—C14C	110.6 (5)	H17D—C17B—H17E	109.5
C12C—C13C—H13G	109.5	C6B—C17B—H17F	109.5
C12C—C13C—H13H	109.5	H17D—C17B—H17F	109.5
H13G—C13C—H13H	109.5	H17E—C17B—H17F	109.5
C12C—C13C—H13I	109.5	C23B—C18B—C19B	118.3 (3)
H13G—C13C—H13I	109.5	C23B—C18B—C3B	121.8 (3)
H13H—C13C—H13I	109.5	C19B—C18B—C3B	119.8 (3)
C12C—C14C—H14G	109.5	C18B—C19B—C20B	120.8 (3)
C12C—C14C—H14H	109.5	C18B—C19B—H19B	119.6
H14G—C14C—H14H	109.5	C20B—C19B—H19B	119.6
C12C—C14C—H14I	109.5	C21B—C20B—C19B	120.9 (3)
H14G—C14C—H14I	109.5	C21B—C20B—H20B	119.6
H14H—C14C—H14I	109.5	C19B—C20B—H20B	119.6
C12C—C15C—H15G	109.5	C20B—C21B—C22B	118.2 (3)
C12C—C15C—H15H	109.5	C20B—C21B—C24B	120.5 (3)
H15G—C15C—H15H	109.5	C22B—C21B—C24B	121.2 (3)
C12C—C15C—H15I	109.5	C23B—C22B—C21B	120.5 (3)
H15G—C15C—H15I	109.5	C23B—C22B—H22B	119.7
H15H—C15C—H15I	109.5	C21B—C22B—H22B	119.7
O1C—C5C—C4C	121.1 (6)	C18B—C23B—C22B	121.3 (3)
O1C—C5C—C6C	118.3 (8)	C18B—C23B—H23B	119.4
C4C—C5C—C6C	119.5 (9)	C22B—C23B—H23B	119.4
C16C—C6C—C17C	109.5 (6)	C29B—C24B—C25B	117.9 (4)
C16C—C6C—C7C	111.1 (5)	C29B—C24B—C21B	120.9 (4)
C17C—C6C—C7C	110.0 (6)	C25B—C24B—C21B	121.1 (4)
C16C—C6C—C5C	108.4 (7)	C26B—C25B—C24B	121.2 (5)
C17C—C6C—C5C	109.4 (7)	C26B—C25B—H25B	119.4
C7C—C6C—C5C	108.4 (6)	C24B—C25B—H25B	119.4
C8C—C7C—C6C	113.2 (8)	C25B—C26B—C27B	119.8 (5)
C8C—C7C—H7CA	108.9	C25B—C26B—H26B	120.1
C6C—C7C—H7CA	108.9	C27B—C26B—H26B	120.1
C8C—C7C—H7CB	108.9	C28B—C27B—C26B	120.0 (4)
C6C—C7C—H7CB	108.9	C28B—C27B—H27B	120.0
H7CA—C7C—H7CB	107.7	C26B—C27B—H27B	120.0
C7C—C8C—C9C	109.1 (8)	C27B—C28B—C29B	119.5 (5)
C7C—C8C—H8CA	109.9	C27B—C28B—H28B	120.2
C9C—C8C—H8CA	109.9	C29B—C28B—H28B	120.2
C7C—C8C—H8CB	109.9	C24B—C29B—C28B	121.6 (5)
C9C—C8C—H8CB	109.9	C24B—C29B—H29B	119.2
H8CA—C8C—H8CB	108.3	C28B—C29B—H29B	119.2
C6C—C16C—H16G	109.5	C11A—O3A—C12A	121.3 (3)
C6C—C16C—H16H	109.5	C9A—N1A—C1A	122.2 (3)
H16G—C16C—H16H	109.5	C9A—N1A—H1NA	113 (3)
C6C—C16C—H16I	109.5	C1A—N1A—H1NA	121 (3)
H16G—C16C—H16I	109.5	C2A—C1A—N1A	119.2 (3)
H16H—C16C—H16I	109.5	C2A—C1A—C10A	128.9 (3)
C6C—C17C—H17G	109.5	N1A—C1A—C10A	111.8 (3)
C6C—C17C—H17H	109.5	C1A—C2A—C11A	124.3 (3)
H17G—C17C—H17H	109.5	C1A—C2A—C3A	120.7 (3)
C6C—C17C—H17I	109.5	C11A—C2A—C3A	115.0 (3)
H17G—C17C—H17I	109.5	C4A—C3A—C2A	110.7 (3)
H17H—C17C—H17I	109.5	C4A—C3A—C18A	112.9 (3)
O1C—C5F—C6F	120 (3)	C2A—C3A—C18A	108.8 (3)
O1C—C5F—C4C	121.6 (19)	C4A—C3A—H3AA	108.1
C6F—C5F—C4C	113 (2)	C2A—C3A—H3AA	108.1
C16F—C6F—C7F	109.8 (14)	C18A—C3A—H3AA	108.1
C16F—C6F—C17F	109.4 (14)	C9A—C4A—C5A	120.4 (3)
C7F—C6F—C17F	112.4 (14)	C9A—C4A—C3A	120.2 (3)
C16F—C6F—C5F	105.7 (17)	C5A—C4A—C3A	119.4 (3)
C7F—C6F—C5F	109.8 (19)	O1A—C5A—C4A	120.6 (3)
C17F—C6F—C5F	109.5 (19)	O1A—C5A—C6A	118.9 (3)
C8F—C7F—C6F	112 (2)	C4A—C5A—C6A	120.5 (3)
C8F—C7F—H7FA	109.3	C17A—C6A—C5A	111.0 (3)
C6F—C7F—H7FA	109.3	C17A—C6A—C16A	110.1 (4)
C8F—C7F—H7FB	109.3	C5A—C6A—C16A	106.5 (3)
C6F—C7F—H7FB	109.3	C17A—C6A—C7A	108.8 (3)
H7FA—C7F—H7FB	107.9	C5A—C6A—C7A	110.7 (3)
C9C—C8F—C7F	119 (3)	C16A—C6A—C7A	109.7 (3)
C9C—C8F—H8FA	107.5	C8A—C7A—C6A	113.2 (3)
C7F—C8F—H8FA	107.5	C8A—C7A—H7AA	108.9
C9C—C8F—H8FB	107.5	C6A—C7A—H7AA	108.9
C7F—C8F—H8FB	107.5	C8A—C7A—H7AB	108.9
H8FA—C8F—H8FB	107.0	C6A—C7A—H7AB	108.9
C6F—C16F—H16J	109.5	H7AA—C7A—H7AB	107.8
C6F—C16F—H16K	109.5	C9A—C8A—C7A	110.3 (3)
H16J—C16F—H16K	109.5	C9A—C8A—H8AA	109.6
C6F—C16F—H16L	109.5	C7A—C8A—H8AA	109.6
H16J—C16F—H16L	109.5	C9A—C8A—H8AB	109.6
H16K—C16F—H16L	109.5	C7A—C8A—H8AB	109.6
C6F—C17F—H17J	109.5	H8AA—C8A—H8AB	108.1
C6F—C17F—H17K	109.5	N1A—C9A—C4A	120.2 (3)
H17J—C17F—H17K	109.5	N1A—C9A—C8A	116.5 (3)
C6F—C17F—H17L	109.5	C4A—C9A—C8A	123.3 (3)
H17J—C17F—H17L	109.5	C1A—C10A—H10A	109.5
H17K—C17F—H17L	109.5	C1A—C10A—H10B	109.5
C23C—C18C—C19C	117.8 (3)	H10A—C10A—H10B	109.5
C23C—C18C—C3C	120.9 (3)	C1A—C10A—H10C	109.5
C19C—C18C—C3C	121.2 (3)	H10A—C10A—H10C	109.5
C20C—C19C—C18C	120.8 (4)	H10B—C10A—H10C	109.5
C20C—C19C—H19C	119.6	O2A—C11A—O3A	124.7 (3)
C18C—C19C—H19C	119.6	O2A—C11A—C2A	122.0 (3)
C21C—C20C—C19C	121.3 (4)	O3A—C11A—C2A	113.3 (3)
C21C—C20C—H20C	119.4	C13A—C12A—O3A	110.1 (4)
C19C—C20C—H20C	119.4	C13A—C12A—C15A	112.6 (5)
C20C—C21C—C22C	117.7 (4)	O3A—C12A—C15A	110.9 (4)
C20C—C21C—C24C	121.0 (4)	C13A—C12A—C14A	111.8 (5)
C22C—C21C—C24C	121.3 (4)	O3A—C12A—C14A	101.0 (3)
C23C—C22C—C21C	121.6 (4)	C15A—C12A—C14A	109.9 (5)
C23C—C22C—H22C	119.2	C12A—C13A—H13A	109.5
C21C—C22C—H22C	119.2	C12A—C13A—H13B	109.5
C22C—C23C—C18C	120.8 (4)	H13A—C13A—H13B	109.5
C22C—C23C—H23C	119.6	C12A—C13A—H13C	109.5
C18C—C23C—H23C	119.6	H13A—C13A—H13C	109.5
C25C—C24C—C29C	118.3 (5)	H13B—C13A—H13C	109.5
C25C—C24C—C21C	121.4 (5)	C12A—C14A—H14A	109.5
C29C—C24C—C21C	120.3 (4)	C12A—C14A—H14B	109.5
C26C—C25C—C24C	121.6 (7)	H14A—C14A—H14B	109.5
C26C—C25C—H25C	119.2	C12A—C14A—H14C	109.5
C24C—C25C—H25C	119.2	H14A—C14A—H14C	109.5
C27C—C26C—C25C	120.1 (6)	H14B—C14A—H14C	109.5
C27C—C26C—H26C	120.0	C12A—C15A—H15A	109.5
C25C—C26C—H26C	120.0	C12A—C15A—H15B	109.5
C26C—C27C—C28C	120.3 (6)	H15A—C15A—H15B	109.5
C26C—C27C—H27C	119.9	C12A—C15A—H15C	109.5
C28C—C27C—H27C	119.9	H15A—C15A—H15C	109.5
C29C—C28C—C27C	119.5 (6)	H15B—C15A—H15C	109.5
C29C—C28C—H28C	120.3	C6A—C16A—H16A	109.5
C27C—C28C—H28C	120.3	C6A—C16A—H16B	109.5
C28C—C29C—C24C	120.2 (6)	H16A—C16A—H16B	109.5
C28C—C29C—H29C	119.9	C6A—C16A—H16C	109.5
C24C—C29C—H29C	119.9	H16A—C16A—H16C	109.5
C2B—C1B—N1B	119.4 (3)	H16B—C16A—H16C	109.5
C2B—C1B—C10B	127.8 (4)	C6A—C17A—H17A	109.5
N1B—C1B—C10B	112.8 (3)	C6A—C17A—H17B	109.5
C11B—O3B—C12B	122.0 (3)	H17A—C17A—H17B	109.5
C9B—N1B—C1B	122.4 (3)	C6A—C17A—H17C	109.5
C9B—N1B—H1NB	121 (2)	H17A—C17A—H17C	109.5
C1B—N1B—H1NB	115 (2)	H17B—C17A—H17C	109.5
C1B—C2B—C11B	125.0 (3)	C23A—C18A—C19A	118.2 (3)
C1B—C2B—C3B	120.2 (3)	C23A—C18A—C3A	121.4 (3)
C11B—C2B—C3B	114.7 (3)	C19A—C18A—C3A	120.3 (3)
C4B—C3B—C2B	110.6 (3)	C20A—C19A—C18A	120.3 (3)
C4B—C3B—C18B	112.0 (3)	C20A—C19A—H19A	119.9
C2B—C3B—C18B	110.5 (3)	C18A—C19A—H19A	119.9
C4B—C3B—H3BA	107.8	C21A—C20A—C19A	121.3 (3)
C2B—C3B—H3BA	107.8	C21A—C20A—H20A	119.3
C18B—C3B—H3BA	107.8	C19A—C20A—H20A	119.3
C9B—C4B—C5B	121.0 (3)	C20A—C21A—C22A	118.1 (3)
C9B—C4B—C3B	120.5 (3)	C20A—C21A—C24A	121.5 (3)
C5B—C4B—C3B	118.4 (3)	C22A—C21A—C24A	120.3 (3)
O1B—C5B—C4B	121.8 (3)	C21A—C22A—C23A	121.1 (3)
O1B—C5B—C6B	118.3 (3)	C21A—C22A—H22A	119.4
C4B—C5B—C6B	119.9 (3)	C23A—C22A—H22A	119.4
C16B—C6B—C7B	110.4 (4)	C18A—C23A—C22A	120.9 (3)
C16B—C6B—C5B	111.1 (3)	C18A—C23A—H23A	119.5
C7B—C6B—C5B	110.8 (3)	C22A—C23A—H23A	119.5
C16B—C6B—C17B	107.6 (4)	C25A—C24A—C29A	118.3 (4)
C7B—C6B—C17B	111.8 (3)	C25A—C24A—C21A	121.5 (4)
C5B—C6B—C17B	105.0 (3)	C29A—C24A—C21A	120.2 (4)
C6B—C7B—C8B	114.0 (3)	C24A—C25A—C26A	120.6 (5)
C6B—C7B—H7BA	108.8	C24A—C25A—H25A	119.7
C8B—C7B—H7BA	108.8	C26A—C25A—H25A	119.7
C6B—C7B—H7BB	108.8	C27A—C26A—C25A	120.3 (5)
C8B—C7B—H7BB	108.8	C27A—C26A—H26A	119.9
H7BA—C7B—H7BB	107.7	C25A—C26A—H26A	119.9
C9B—C8B—C7B	110.4 (3)	C26A—C27A—C28A	119.8 (4)
C9B—C8B—H8BA	109.6	C26A—C27A—H27A	120.1
C7B—C8B—H8BA	109.6	C28A—C27A—H27A	120.1
C9B—C8B—H8BB	109.6	C27A—C28A—C29A	120.7 (5)
C7B—C8B—H8BB	109.6	C27A—C28A—H28A	119.7
H8BA—C8B—H8BB	108.1	C29A—C28A—H28A	119.7
C4B—C9B—N1B	120.5 (3)	C28A—C29A—C24A	120.3 (5)
C4B—C9B—C8B	123.4 (3)	C28A—C29A—H29A	119.9
N1B—C9B—C8B	116.0 (3)	C24A—C29A—H29A	119.9
C1B—C10B—H10D	109.5		
			
C9C—N1C—C1C—C2C	−13.3 (5)	C5B—C6B—C7B—C8B	−49.0 (5)
C9C—N1C—C1C—C10C	165.7 (3)	C17B—C6B—C7B—C8B	67.8 (5)
N1C—C1C—C2C—C11C	167.0 (3)	C6B—C7B—C8B—C9B	48.9 (5)
C10C—C1C—C2C—C11C	−11.9 (6)	C5B—C4B—C9B—N1B	171.9 (3)
N1C—C1C—C2C—C3C	−11.3 (5)	C3B—C4B—C9B—N1B	−6.1 (5)
C10C—C1C—C2C—C3C	169.8 (4)	C5B—C4B—C9B—C8B	−5.5 (5)
C1C—C2C—C3C—C18C	−94.9 (4)	C3B—C4B—C9B—C8B	176.5 (3)
C11C—C2C—C3C—C18C	86.6 (4)	C1B—N1B—C9B—C4B	−13.2 (5)
C1C—C2C—C3C—C4C	29.8 (4)	C1B—N1B—C9B—C8B	164.3 (3)
C11C—C2C—C3C—C4C	−148.6 (3)	C7B—C8B—C9B—C4B	−21.2 (5)
C18C—C3C—C4C—C9C	96.0 (4)	C7B—C8B—C9B—N1B	161.3 (3)
C2C—C3C—C4C—C9C	−27.9 (5)	C12B—O3B—C11B—O2B	−6.2 (6)
C18C—C3C—C4C—C5C	−83.8 (7)	C12B—O3B—C11B—C2B	172.5 (4)
C2C—C3C—C4C—C5C	152.3 (7)	C1B—C2B—C11B—O2B	162.4 (4)
C18C—C3C—C4C—C5F	−76.5 (17)	C3B—C2B—C11B—O2B	−14.1 (5)
C2C—C3C—C4C—C5F	159.6 (16)	C1B—C2B—C11B—O3B	−16.4 (6)
C5C—C4C—C9C—N1C	−172.9 (7)	C3B—C2B—C11B—O3B	167.1 (3)
C3C—C4C—C9C—N1C	7.3 (5)	C11B—O3B—C12B—C15B	62.0 (6)
C5F—C4C—C9C—N1C	179.1 (18)	C11B—O3B—C12B—C13B	−63.1 (5)
C3C—C4C—C9C—C8F	−173 (3)	C11B—O3B—C12B—C14B	−179.9 (4)
C5F—C4C—C9C—C8F	−1 (4)	C4B—C3B—C18B—C23B	46.4 (4)
C5C—C4C—C9C—C8C	6.9 (14)	C2B—C3B—C18B—C23B	−77.4 (4)
C3C—C4C—C9C—C8C	−172.9 (11)	C4B—C3B—C18B—C19B	−137.8 (3)
C1C—N1C—C9C—C4C	15.4 (5)	C2B—C3B—C18B—C19B	98.3 (4)
C1C—N1C—C9C—C8F	−164 (3)	C23B—C18B—C19B—C20B	0.0 (5)
C1C—N1C—C9C—C8C	−164.4 (10)	C3B—C18B—C19B—C20B	−175.9 (3)
C12C—O3C—C11C—O2C	3.1 (6)	C18B—C19B—C20B—C21B	0.4 (6)
C12C—O3C—C11C—C2C	−174.1 (4)	C19B—C20B—C21B—C22B	−0.1 (6)
C1C—C2C—C11C—O2C	−175.7 (4)	C19B—C20B—C21B—C24B	177.7 (4)
C3C—C2C—C11C—O2C	2.7 (6)	C20B—C21B—C22B—C23B	−0.5 (6)
C1C—C2C—C11C—O3C	1.6 (6)	C24B—C21B—C22B—C23B	−178.4 (4)
C3C—C2C—C11C—O3C	−180.0 (3)	C19B—C18B—C23B—C22B	−0.7 (6)
C11C—O3C—C12C—C15C	−66.1 (5)	C3B—C18B—C23B—C22B	175.2 (3)
C11C—O3C—C12C—C13C	58.8 (5)	C21B—C22B—C23B—C18B	0.9 (6)
C11C—O3C—C12C—C14C	176.2 (4)	C20B—C21B—C24B—C29B	46.5 (6)
C9C—C4C—C5C—O1C	176.3 (8)	C22B—C21B—C24B—C29B	−135.7 (4)
C3C—C4C—C5C—O1C	−3.9 (14)	C20B—C21B—C24B—C25B	−131.0 (5)
C9C—C4C—C5C—C6C	−15.8 (12)	C22B—C21B—C24B—C25B	46.9 (6)
C3C—C4C—C5C—C6C	164.0 (6)	C29B—C24B—C25B—C26B	−0.2 (8)
O1C—C5C—C6C—C16C	87.0 (11)	C21B—C24B—C25B—C26B	177.3 (5)
C4C—C5C—C6C—C16C	−81.2 (9)	C24B—C25B—C26B—C27B	−0.6 (9)
O1C—C5C—C6C—C17C	−32.3 (12)	C25B—C26B—C27B—C28B	1.1 (9)
C4C—C5C—C6C—C17C	159.4 (8)	C26B—C27B—C28B—C29B	−0.7 (8)
O1C—C5C—C6C—C7C	−152.3 (9)	C25B—C24B—C29B—C28B	0.7 (7)
C4C—C5C—C6C—C7C	39.5 (11)	C21B—C24B—C29B—C28B	−176.9 (4)
C16C—C6C—C7C—C8C	62.9 (10)	C27B—C28B—C29B—C24B	−0.2 (7)
C17C—C6C—C7C—C8C	−175.6 (9)	C9A—N1A—C1A—C2A	−13.7 (5)
C5C—C6C—C7C—C8C	−56.1 (11)	C9A—N1A—C1A—C10A	166.0 (3)
C6C—C7C—C8C—C9C	47.6 (15)	N1A—C1A—C2A—C11A	168.8 (3)
C4C—C9C—C8C—C7C	−22.9 (18)	C10A—C1A—C2A—C11A	−10.8 (6)
N1C—C9C—C8C—C7C	156.9 (8)	N1A—C1A—C2A—C3A	−8.3 (5)
C9C—C4C—C5F—O1C	165 (2)	C10A—C1A—C2A—C3A	172.1 (3)
C3C—C4C—C5F—O1C	−23 (4)	C1A—C2A—C3A—C4A	25.7 (4)
C9C—C4C—C5F—C6F	12 (3)	C11A—C2A—C3A—C4A	−151.7 (3)
C3C—C4C—C5F—C6F	−175.9 (17)	C1A—C2A—C3A—C18A	−99.0 (4)
O1C—C5F—C6F—C16F	50 (3)	C11A—C2A—C3A—C18A	83.6 (4)
C4C—C5F—C6F—C16F	−156 (2)	C2A—C3A—C4A—C9A	−24.3 (4)
O1C—C5F—C6F—C7F	169 (3)	C18A—C3A—C4A—C9A	98.0 (4)
C4C—C5F—C6F—C7F	−37 (3)	C2A—C3A—C4A—C5A	155.9 (3)
O1C—C5F—C6F—C17F	−67 (3)	C18A—C3A—C4A—C5A	−81.7 (4)
C4C—C5F—C6F—C17F	86 (3)	C9A—C4A—C5A—O1A	176.7 (3)
C16F—C6F—C7F—C8F	170 (3)	C3A—C4A—C5A—O1A	−3.5 (5)
C17F—C6F—C7F—C8F	−68 (3)	C9A—C4A—C5A—C6A	−4.4 (5)
C5F—C6F—C7F—C8F	54 (3)	C3A—C4A—C5A—C6A	175.4 (3)
C4C—C9C—C8F—C7F	18 (6)	O1A—C5A—C6A—C17A	−33.9 (5)
N1C—C9C—C8F—C7F	−162 (3)	C4A—C5A—C6A—C17A	147.2 (4)
C6F—C7F—C8F—C9C	−46 (5)	O1A—C5A—C6A—C16A	85.9 (4)
C2C—C3C—C18C—C23C	74.7 (4)	C4A—C5A—C6A—C16A	−93.1 (4)
C4C—C3C—C18C—C23C	−48.2 (4)	O1A—C5A—C6A—C7A	−154.9 (3)
C2C—C3C—C18C—C19C	−100.8 (4)	C4A—C5A—C6A—C7A	26.2 (5)
C4C—C3C—C18C—C19C	136.3 (3)	C17A—C6A—C7A—C8A	−171.9 (3)
C23C—C18C—C19C—C20C	−0.1 (5)	C5A—C6A—C7A—C8A	−49.6 (4)
C3C—C18C—C19C—C20C	175.6 (3)	C16A—C6A—C7A—C8A	67.6 (4)
C18C—C19C—C20C—C21C	−1.6 (6)	C6A—C7A—C8A—C9A	50.7 (4)
C19C—C20C—C21C—C22C	1.7 (6)	C1A—N1A—C9A—C4A	15.1 (5)
C19C—C20C—C21C—C24C	−177.8 (4)	C1A—N1A—C9A—C8A	−165.3 (3)
C20C—C21C—C22C—C23C	−0.2 (6)	C5A—C4A—C9A—N1A	−174.5 (3)
C24C—C21C—C22C—C23C	179.3 (4)	C3A—C4A—C9A—N1A	5.7 (5)
C21C—C22C—C23C—C18C	−1.5 (6)	C5A—C4A—C9A—C8A	5.9 (5)
C19C—C18C—C23C—C22C	1.6 (5)	C3A—C4A—C9A—C8A	−173.9 (3)
C3C—C18C—C23C—C22C	−174.1 (3)	C7A—C8A—C9A—N1A	151.4 (3)
C20C—C21C—C24C—C25C	34.4 (6)	C7A—C8A—C9A—C4A	−29.0 (5)
C22C—C21C—C24C—C25C	−145.0 (5)	C12A—O3A—C11A—O2A	5.4 (6)
C20C—C21C—C24C—C29C	−147.3 (4)	C12A—O3A—C11A—C2A	−173.6 (3)
C22C—C21C—C24C—C29C	33.3 (6)	C1A—C2A—C11A—O2A	−163.6 (4)
C29C—C24C—C25C—C26C	−0.2 (9)	C3A—C2A—C11A—O2A	13.6 (5)
C21C—C24C—C25C—C26C	178.1 (5)	C1A—C2A—C11A—O3A	15.4 (5)
C24C—C25C—C26C—C27C	−1.0 (10)	C3A—C2A—C11A—O3A	−167.3 (3)
C25C—C26C—C27C—C28C	1.5 (10)	C11A—O3A—C12A—C13A	60.1 (6)
C26C—C27C—C28C—C29C	−0.8 (9)	C11A—O3A—C12A—C15A	−65.2 (5)
C27C—C28C—C29C—C24C	−0.5 (8)	C11A—O3A—C12A—C14A	178.3 (5)
C25C—C24C—C29C—C28C	0.9 (7)	C4A—C3A—C18A—C23A	−44.9 (5)
C21C—C24C—C29C—C28C	−177.4 (4)	C2A—C3A—C18A—C23A	78.5 (4)
C2B—C1B—N1B—C9B	11.4 (5)	C4A—C3A—C18A—C19A	140.0 (3)
C10B—C1B—N1B—C9B	−167.1 (3)	C2A—C3A—C18A—C19A	−96.6 (4)
N1B—C1B—C2B—C11B	−166.7 (3)	C23A—C18A—C19A—C20A	−0.1 (5)
C10B—C1B—C2B—C11B	11.5 (6)	C3A—C18A—C19A—C20A	175.1 (3)
N1B—C1B—C2B—C3B	9.6 (5)	C18A—C19A—C20A—C21A	−1.0 (6)
C10B—C1B—C2B—C3B	−172.2 (4)	C19A—C20A—C21A—C22A	1.1 (6)
C1B—C2B—C3B—C4B	−25.4 (4)	C19A—C20A—C21A—C24A	−177.8 (4)
C11B—C2B—C3B—C4B	151.2 (3)	C20A—C21A—C22A—C23A	−0.2 (6)
C1B—C2B—C3B—C18B	99.2 (4)	C24A—C21A—C22A—C23A	178.8 (4)
C11B—C2B—C3B—C18B	−84.1 (4)	C19A—C18A—C23A—C22A	1.0 (6)
C2B—C3B—C4B—C9B	23.8 (4)	C3A—C18A—C23A—C22A	−174.1 (4)
C18B—C3B—C4B—C9B	−100.0 (4)	C21A—C22A—C23A—C18A	−0.9 (6)
C2B—C3B—C4B—C5B	−154.2 (3)	C20A—C21A—C24A—C25A	140.7 (4)
C18B—C3B—C4B—C5B	82.0 (4)	C22A—C21A—C24A—C25A	−38.3 (6)
C9B—C4B—C5B—O1B	−172.8 (3)	C20A—C21A—C24A—C29A	−38.7 (6)
C3B—C4B—C5B—O1B	5.1 (5)	C22A—C21A—C24A—C29A	142.3 (4)
C9B—C4B—C5B—C6B	5.4 (5)	C29A—C24A—C25A—C26A	1.2 (7)
C3B—C4B—C5B—C6B	−176.6 (3)	C21A—C24A—C25A—C26A	−178.2 (5)
O1B—C5B—C6B—C16B	−36.6 (5)	C24A—C25A—C26A—C27A	−1.2 (8)
C4B—C5B—C6B—C16B	145.1 (4)	C25A—C26A—C27A—C28A	−0.1 (9)
O1B—C5B—C6B—C7B	−159.7 (3)	C26A—C27A—C28A—C29A	1.3 (8)
C4B—C5B—C6B—C7B	22.0 (5)	C27A—C28A—C29A—C24A	−1.3 (7)
O1B—C5B—C6B—C17B	79.4 (4)	C25A—C24A—C29A—C28A	0.0 (7)
C4B—C5B—C6B—C17B	−98.9 (4)	C21A—C24A—C29A—C28A	179.4 (4)
C16B—C6B—C7B—C8B	−172.5 (4)		

### Hydrogen-bond geometry (Å, º)

*Cg*4, *Cg*8, *Cg*12 and *Cg*13 are the centroids of the C18*C*–C23*C*, C18*A*–C23*A*, C18*B*–C23*B*, and C18*B*–C29*B* rings, respectively.

**Table:**

*D*—H···*A*	*D*—H	H···*A*	*D*···*A*	*D*—H···*A*
N1*C*—H1*NC*···O1*B*^i^	0.92 (5)	1.94 (5)	2.843 (4)	165 (5)
N1*B*—H1*NB*···O1*C*	0.95 (4)	1.88 (4)	2.811 (4)	168 (3)
N1*A*—H1*NA*···O1*A*^ii^	0.93 (5)	1.92 (5)	2.842 (4)	174 (4)
C3*C*—H3*CA*···O2*C*	1.00	2.41	2.793 (4)	102
C10*A*—H10*B*···O1*A*^ii^	0.98	2.60	3.443 (5)	145
C10*B*—H10*F*···O1*C*	0.98	2.47	3.302 (5)	143
C10*C*—H10*G*···O3*C*	0.98	2.31	2.704 (4)	103
C3*B*—H3*BA*···O2*B*	1.00	2.40	2.795 (4)	103
C13*A*—H13*A*···O2*A*	0.98	2.45	2.978 (7)	113
C13*B*—H13*D*···O2*B*	0.98	2.47	3.023 (6)	115
C13*C*—H13*G*···O2*C*	0.98	2.53	2.958 (7)	106
C15*A*—H15*A*···O2*A*	0.98	2.41	2.999 (7)	118
C15*B*—H15*D*···O2*B*	0.98	2.41	2.965 (6)	116
C15*C*—H15*G*···O2*C*	0.98	2.49	3.022 (5)	114
C3*A*—H3*AA*···O2*A*	1.00	2.42	2.812 (5)	103
C23*A*—H23*A*···O2*A*^ii^	0.95	2.57	3.403 (4)	147
C23*B*—H23*B*···O2*C*	0.95	2.55	3.389 (5)	147
C23*C*—H23*C*···O2*B*^i^	0.95	2.60	3.407 (4)	144
C15*A*—H15*B*···*Cg*13	0.98	2.82	3.771 (6)	165
C27*A*—H27*A*···*Cg*4^ii^	0.95	2.75	3.578 (4)	146
C27*B*—H27*B*···*Cg*8	0.95	2.63	3.493 (5)	150
C27*C*—H27*C*···*Cg*12^iii^	0.95	2.84	3.632 (7)	142

Symmetry codes: (i) *x*, *y*, *z*−1; (ii) −*x*+1/2, *y*, *z*+1/2; (iii) −*x*+1/2, *y*, *z*−1/2.
